# Zoonotic pathogens in wild Asian primates: a systematic review highlighting research gaps

**DOI:** 10.3389/fvets.2024.1386180

**Published:** 2024-06-27

**Authors:** Laurie Patouillat, Alain Hambuckers, Sena Adi Subrata, Mutien Garigliany, Fany Brotcorne

**Affiliations:** ^1^SPHERES, Primatology and Tropical Ecology Group, Faculty of Sciences, University of Liège, Liège, Belgium; ^2^FARAH, Department of Pathology, Faculty of Veterinary Medicine, University of Liège, Liège, Belgium; ^3^Faculty of Forestry, Universitas Gadjah Mada, Yogyakarta, Indonesia

**Keywords:** human-primate interface, habitat type, infection diagnostics, pathogen specific richness, gastrointestinal parasites, virus, bacteria, protozoa

## Abstract

**Introduction:**

Ongoing global changes, including natural land conversion for agriculture and urbanization, modify the dynamics of human–primate contacts, resulting in increased zoonotic risks. Although Asia shelters high primate diversity and experiences rapid expansion of human–primate contact zones, there remains little documentation regarding zoonotic surveillance in the primates of this region.

**Methods:**

Using the PRISMA guidelines, we conducted a systematic review to compile an inventory of zoonotic pathogens detected in wild Asian primates, while highlighting the coverage of primate species, countries, and pathogen groups surveyed, as well as the diagnostic methods used across the studies. Moreover, we compared the species richness of pathogens harbored by primates across diverse types of habitats classified according to their degree of anthropization (i.e., urban vs. rural vs. forest habitats).

**Results and discussion:**

Searches of Scopus, PubMed, and the Global Mammal Parasite Database yielded 152 articles on 39 primate species. We inventoried 183 pathogens, including 63 helminthic gastrointestinal parasites, two blood-borne parasites, 42 protozoa, 45 viruses, 30 bacteria, and one fungus. Considering each study as a sample, species accumulation curves revealed no significant differences in specific richness between habitat types for any of the pathogen groups analyzed. This is likely due to the insufficient sampling effort (i.e., a limited number of studies), which prevents drawing conclusive findings. This systematic review identified several publication biases, particularly the uneven representation of host species and pathogen groups studied, as well as a lack of use of generic diagnostic methods. Addressing these gaps necessitates a multidisciplinary strategy framed in a One Health approach, which may facilitate a broader inventory of pathogens and ultimately limit the risk of cross-species transmission at the human–primate interface. Strengthening the zoonotic surveillance in primates of this region could be realized notably through the application of more comprehensive diagnostic techniques such as broad-spectrum analyses without *a priori* selection.

## 1 Introduction

The expansion of human populations, coupled with natural habitat degradation, land-use change, and illegal hunting, have broken down the natural barriers between humans and non-human primates (hereafter, primates), forcing the latter to increasingly live in human-modified environments (1, 2). Even though humans have always shared habitats with primates in some regions, the dynamics of human–primate interactions are radically changing and intensifying. As a result, increasing contacts and conflicts occur, representing a growing risk for zoonotic transmission and wildlife conservation (3, 4). More specifically, the zoonotic risk increases with changes in the dynamics of interactions following (i) the loss and fragmentation of natural habitats for agricultural and industrialization purposes, (ii) the expansion of road networks, and (iii) the greater urban demands for bushmeat and exotic pets, which exacerbate wildlife exploitation (1, 5, 6). Zoonotic pathogens can be transmitted naturally from vertebrate animals to humans, as opposed to reverse zoonotic agents, which are transmitted from humans to animals (7). A large proportion of the major human infectious diseases like measles, plague, or yellow fever, originate in animals, notably in domestic animals within temperate regions, or in non-human primates, the closest evolutionary relatives to humans, found in tropical regions (8). Nowadays, pathogens can spread more rapidly to new regions through international travel and commerce, and be transmitted to new susceptible hosts (9). The alteration of historical distribution patterns of pathogens, associated with the increasing spatial proximity between species, has allowed novel species to come into contact with new specific infectious agents, thus increasing the risk of epidemics (10, 11).

Emerging infectious diseases (EIDs), of which ~75% are of animal origin and are caused by infectious organisms, are characterized by a very large increase of new infections in a host population over a given period of time. This surge in infections likely leads to epidemics or pandemics (12). Recent epidemics such as coronavirus disease 2019 (COVID-19), caused by severe acute respiratory syndrome coronavirus 2 (SARS-CoV-2), and the Ebola virus disease, have stressed how knowledge about human–animal interactions and ecosystem health is essential to control the emergence and spread of zoonotic diseases (13). Thus, it is necessary to assess the factors influencing the risks for human and animal health (14). The process of disease emergence appears to be driven by recent changes in human demographics and behavior and by ecological disruption (15, 16). While habitat alteration typically leads to biodiversity loss (17, 18), the risk of pathogen transmission is strongly linked to the diversity of host species in an ecosystem (19). More specifically, the likelihood of the emergence of zoonotic pathogens depends on several factors, including the prevalence of zoonotic pathogens in wildlife reservoirs, the frequency and intensity of interspecies contacts, the effects of environmental changes on these reservoirs and vectors (e.g., modified geographic range of diseases following climate change), and the type of habitat (6, 20, 21). Currently, the influence of habitat degradation on the prevalence and diversity of infectious pathogens in wildlife is still debated, with conflicting findings reported (22, 23). Some studies have shown that habitat anthropization is negatively correlated with wildlife health, while other studies support that urban environments have no negative effect or even positive effects on animal health. Regarding primates, a study on parasite infection in toque macaques (*Macaca sinica*) and lion-tailed macaques (*Macaca silenus*) (24) living in (sub)urban habitats showed a higher parasite richness and prevalence compared with the populations living in undisturbed natural habitats. Conversely, in long-tailed macaques (*Macaca fascicularis*), it was found a lower prevalence and diversity of gastrointestinal (GI) parasites and protozoa in anthropogenic landscapes (25).

Two contrasting concepts in disease ecology describe the influence of biodiversity loss on the prevalence of pathogens in an ecosystem. The dilution effect implies that biodiversity may act as a barrier to the spread of infectious diseases. Indeed, in a diverse ecosystem, high species diversity may dilute the pool of host species that are competent for pathogens, including many poor reservoirs, thus reducing the persistence and transmission of some pathogens (26). Conversely, the amplification effect represents the scenario in which high biodiversity with diverse competent zoonotic reservoirs or vectors promotes the prevalence of more diverse pathogens and their transmission to humans (27). The predominance of those effects depends on many ecological factors, including the host community and the specific diseases. Nonetheless, it has been suggested that some synanthropic animal species that proliferate in human-dominated environments are more likely to be competent hosts for EIDs than others and, therefore, increase the risk of pathogen transmission to humans (28). Conversely, in less disturbed habitats, competent zoonotic reservoir hosts are less prevalent, and non-reservoir species predominate. Therefore, biodiversity loss in human-modified environments appears to increase the risk of human exposure to new or established zoonotic pathogens (26).

Humans tend to share a greater proportion of pathogens with primates compared with others animals, due to their genetic, physiological, and sometimes social similarities (29). Ebola virus and human immunodeficiency virus (HIV) are textbook examples of epidemic viruses that originated from primates (30–32). These epidemics illustrate how primates can be potential reservoirs of zoonotic infectious agents (33, 34). Zoonotic pathogens (viruses, bacteria, parasites, and fungi) can be transmitted between primates and humans via (in)direct contacts and several pathways (30). They may spread rapidly via direct host-to-host contacts (e.g., respiratory viruses) or by exchange of body fluids such as blood, urine, or saliva (e.g., herpesvirus B and simian foamy virus). GI parasites may enter hosts via exposure to shared contaminated environmental sources such as food, water, and soil. Pathogens can also be transmitted by vectors such as arthropods (e.g., *Plasmodium knowlesi* transmitted through the mosquitoes *Anopheles latens* and *A. hackeri*) (35).

The ongoing biodiversity crisis has taught us that primates are a particularly vulnerable group. Two thirds of primate species are threatened with extinction mostly due to anthropogenic pressures driving habitat loss, species exploitation, and emerging threats including zoonotic diseases (34, 36, 37). A recent interest has developed regarding the transmission mechanisms and the prevalence of zoonotic pathogens in primates that interact with humans (13, 38). The term “human–primate interface” encapsulates all aspects of the socio-ecological relationships linking humans and other primates together, that is, their dynamic interactions in shared environments (39). This interface is diverse. There are different degrees of habitat anthropization, such as urban settings, rural landscapes, and forest habitats, where multiple social and environmental factors may influence the likelihood of interspecies transmission of zoonotic pathogens (22, 40). Therefore, adopting a One Health transdisciplinary approach by recognizing the interconnected links between human, animal, and environmental health is particularly relevant in such interfaces (11, 41).

Asia represents a critical hotspot for zoonotic EIDs (42) given the high human population density combined with a large primate community and frequent human–primate contacts (28). Especially in South and Southeast Asia, humans and primates increasingly overlap spatially and ecologically in cities, temples, and recreational parks. Generalist primate species such as the rhesus macaque (*Macaca mulatta*) or the long-tailed macaque (*M. fascicularis*) are synanthropic species frequently encountered in anthropogenic habitats due to their ecological and behavioral flexibility (2, 43). Notwithstanding the critical significance of the cross-species transmission risk at the human–primate interface (44), lacunae persist in the comprehension of host–pathogen dynamics and the compilation of zoonotic pathogens across primate species, notably within specific regions such as Southeast Asia (45). Few studies have sought to review zoonotic pathogens detected in free-ranging Asian primates. Balasubramaniam et al. (40) focused on gastrointestinal parasites in Asian macaques. In another recent review, Liu et al. (46) surveyed viral infections among primates worldwide, encompassing studies involving both captive and wild individuals. Yet, a holistic review requires the inclusion of diverse types of pathogens and all Asian primate species. In addition, there is still a need to better understand how the risk of infections is influenced by environmental factors, such as the type of habitat.

Thus, the primary objective of this systematic review is to compile an inventory of the zoonotic pathogens reported from free-ranging Asian primates, exploring the diversity of pathogens found across diverse habitat types (i.e., forest vs. rural vs. urban habitat) and their routes of transmission. Through this updated overview, we aim to investigating potential disparities in the current knowledge about pathogen groups surveyed among primate species, Asian countries, and diagnostic methods employed.

## 2 Methods

### 2.1 Data compilation

**Figure 1:**
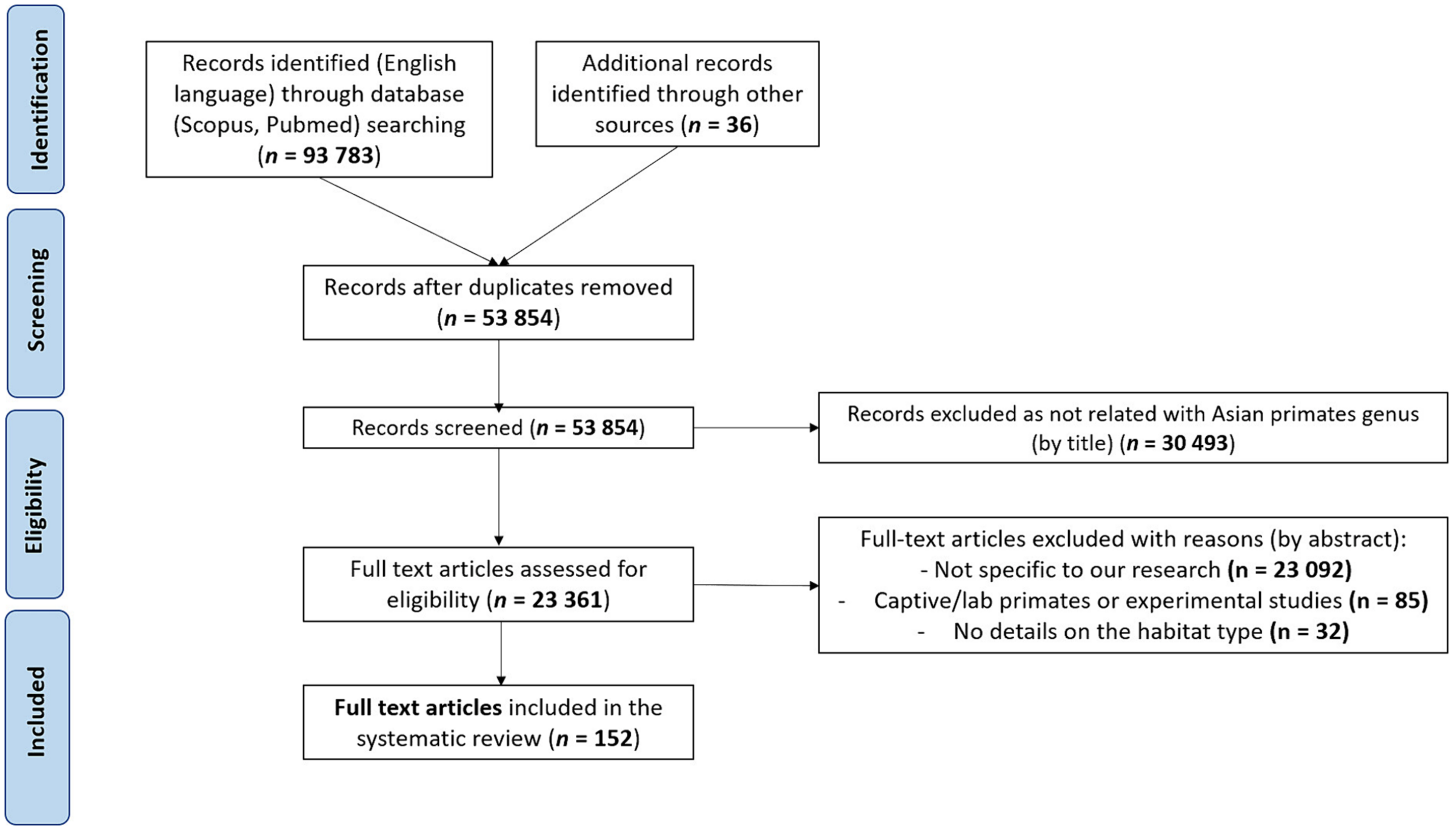
Preferred Reporting Items for Systematic Reviews and Meta-Analyses (PRISMA) flowchart describing the literature search and the selection of articles.

Using the Preferred Reporting Items for Systematic Reviews and Meta-Analyses (PRISMA) methods, we conducted a systematic literature search of papers up to December 2023 on zoonotic pathogens (i.e., bacteria, protozoa, viruses, fungi, and metazoan parasites) found in non-captive primates living in and native to Asia. Because our goal was to inventory zoonotic pathogens in free-ranging Asian primates and living in different habitat types, we did not include studies on captive or laboratory primates. We searched Scopus, PubMed, and the Global Mammal Parasite Database (47) with the following keywords and Boolean operators in abstract, title and keywords: “primate* OR monkey* AND pathogen* OR disease* OR zoono* OR infect* AND virus* OR parasite* OR bacteria OR fungi AND NOT captive* OR experimental OR zoo AND NOT Afric* OR Neotropic* AND NOT chimpanzee* OR gorilla* OR capuchin* OR baboon*.” We also included additional records identified through other sources (based on article reading or the reference lists of the included studies). After identifying the articles, we screened them by eliminating studies using the following exclusion criteria: (1) The study was not performed on wild primates and native to Asia (papers included referred specifically to primate species ranging in three regions: South Asia, East Asia, and Southeast Asia). (2) The study did not search for at least one zoonotic pathogen (a pathogen was considered to be zoonotic if it is explicitly defined as zoonotic in the article or if it has been listed at least once as infecting humans in the literature). (3) The study did not provide information on the habitat type where the screened primates lived. (4) The study was written in a language other than English. (5) The study was a duplicate, not an original research article or reported same database. As for eligibility, we included all records that clearly indicated the species of the zoonotic pathogen, the host species, and the type of habitat where the host lives (Figure 1). In the end, we included a total of 152 studies in this review.

For each included study, we recorded the following information: (1) primate host species, (2) zoonotic pathogen taxa recorded, (3) main pathogen transmission route (i.e., respiratory, body fluid contact, vector borne, and fecal-oral route, determined according to the literature), (4) country where the study was performed, (5) type of habitat in which the host lives, and (6) diagnostic methods used to identify zoonotic pathogens. We distinguished between generic detection methods, which include microscopy, metagenomics, spectrometry and culture; and specific detection methods which encompass polymerase chain reaction (PCR), sequencing, serology, and isolation. As regard microscopy, our typology included three categories: (a) direct optic examination with staining, (b) direct optic examination without staining (only flotation and/or sedimentation), and (c) direct electron microscopy examination. Finally, we divided the zoonotic pathogens into six groups: viruses, fungi, bacteria, protozoa, and gastrointestinal metazoan parasites (hereafter, GI parasites) including Platyhelminthes (Cestoda and Trematoda) and Nematoda, and blood-borne parasites.

To compare pathogen diversity between habitat types, we classified the studies according to the degree of anthropization of the habitat in which the primates live. This level of anthropization was determined by relying on the habitat descriptions provided in the articles. Based on a simple system of landscape classification according to anthropogenic disturbance and land use, we distinguished between urban, rural, and forest habitats (48). Urban habitats, characterized by the highest anthropization degree, are defined as zones where human infrastructures prevail, such as towns, villages, temples, and gardens. Urban habitats are also characterized by the highest degree of spatial overlap between humans and primates. Rural habitats correspond to an intermediate degree of anthropization, including open areas (cropland and pastures), tree plantations, agroforestry, and small villages. In rural habitats, crop-feeding by primates is frequently observed. Finally, forest habitats include secondary forests that have undergone human disturbances such as fragmentation or logging, and more preserved forests in protected areas where human impact remains limited. Forest habitats have the least spatial overlap between humans and primates, and primates mainly feed on natural resources.

### 2.2 Data analysis

When a single study investigated several elements belonging to the same variable of interest (i.e., different taxa of zoonotic pathogens, different types of habitats, different species of host primates, different types of transmission routes, or different types of diagnostic methods), we considered each element as a separate study in the analysis. For example, we counted a study having screened protozoa and GI parasites as two separate studies in the analysis.

We used extrapolation of accumulation curves of species richness (49, 50) to quantify and statistically measure the differences across habitat types in the diversity of zoonotic agent species, while accounting for uneven sampling efforts (50). We used sample-based species accumulation curves to model the rarefaction curves, that is, the expectation of the cumulative number of species for a given number of samples. The extrapolation of the rarefaction curve is based on a Bernoulli product model including a non-parametric estimator of total species richness and provides exhaustive species richness and confidence intervals (51). We performed this procedure for each type of habitat and each group of pathogens using the EstimateSWin.8.2 software (52). Due to limited number of studies for fungi (*N* = 3 studies) and blood-borne parasites (*N* = 1 study), we only conducted this analysis for GI parasites (*N* = 57 studies), protozoa (*N* = 68 studies), bacteria (*N* = 23 studies), and viruses (*N* = 35 studies).

## 3 Results

### 3.1 Overview of zoonotic pathogen reported in Asian primates

#### 3.1.1 Sampling effort by country

**Figure 2:**
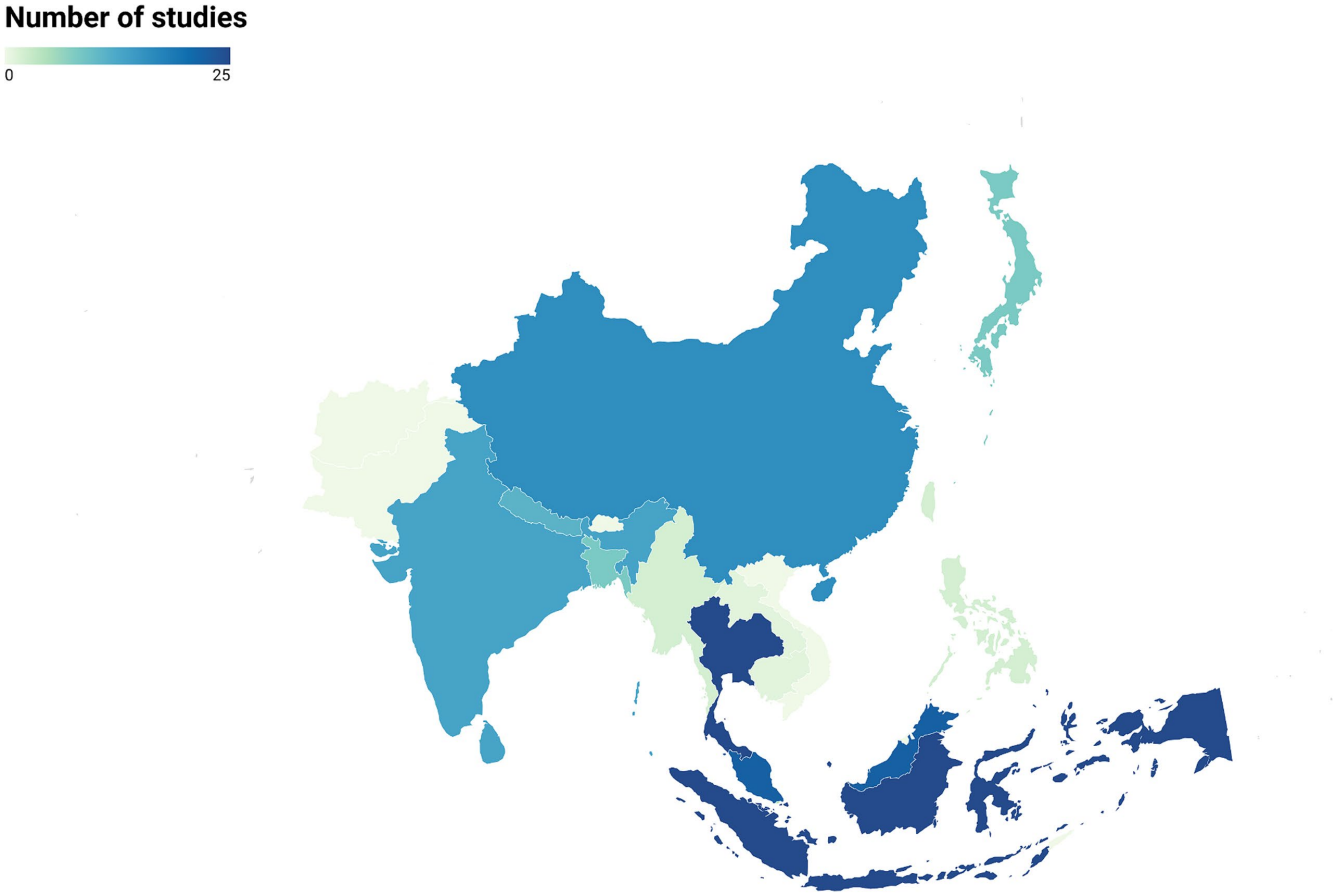
Distribution of studies on zoonotic pathogens in free-ranging primates across Asian countries (*N* = 152 articles).

Our search of the literature yielded 152 articles dating from 1965 to 2023 that studied zoonotic pathogens in free-ranging primates living in 15 Asian countries (Figure 2). Thailand and Indonesia were the countries with the highest number of studies (*N* = 25 for each). Laos and Cambodia had the lowest number of studies (*N* = 1 for each country). Finally, there were no studies for several primate-range Asian countries, including Pakistan, Bhutan, Afghanistan, Timor-Leste, and Vietnam (Figure 2).

#### 3.1.2 Primate species

**Figure 3:**
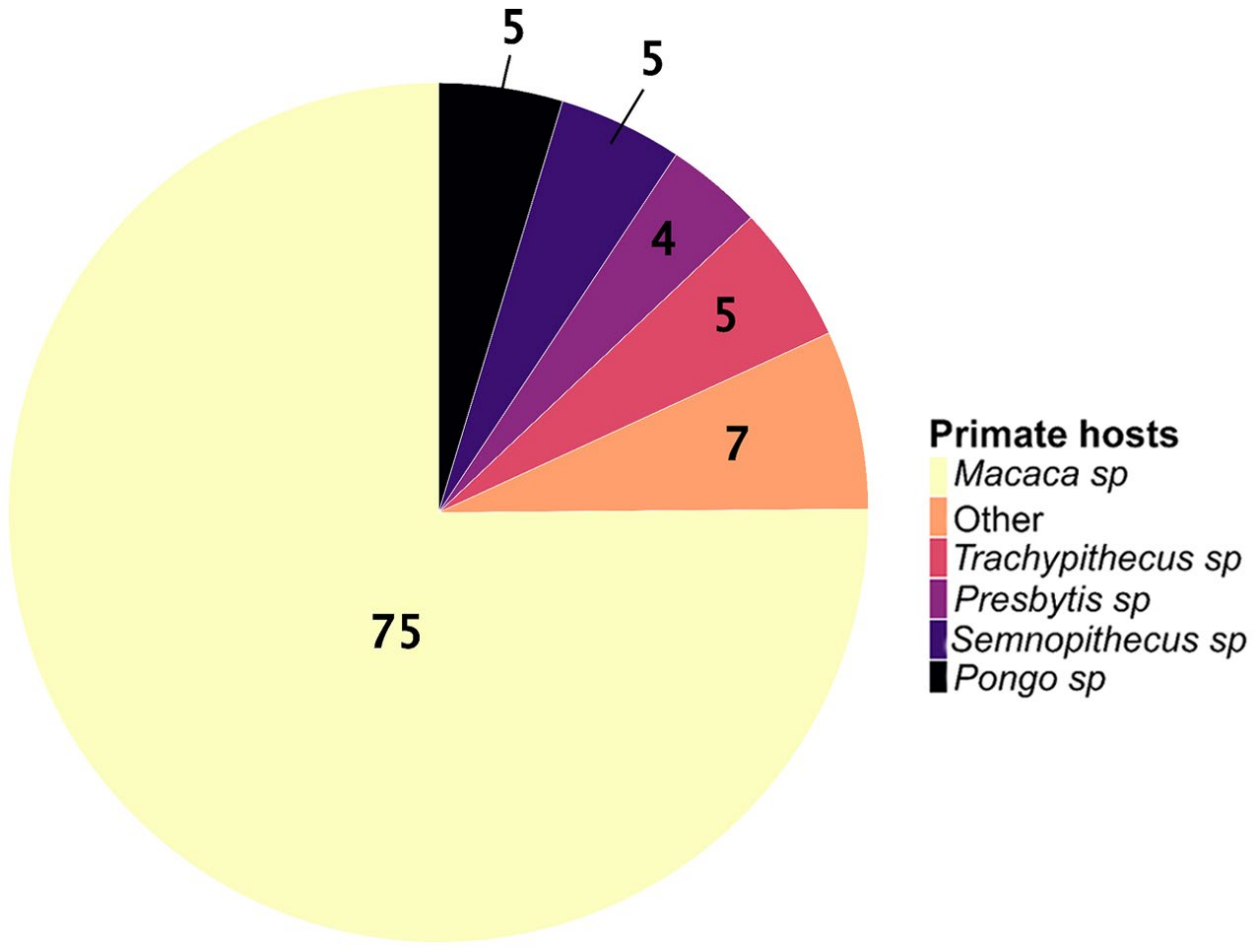
The proportions of wild Asian primate genera studied for zoonotic pathogens (*N* = 193 studies). The “other” category corresponds to the sum of the proportions for the genera *Nasalis* sp., *Hylobates* sp., *Cephalopachus* sp., *Nycticebus* sp., *Rhinopithecus* sp., and *Tarsius* sp.

We identified an uneven distribution of studies on zoonotic pathogens across primate genera and species. Although 119 species of primates, belonging to 18 genera, are found in Asia (34), only 11 genera (61%) have been screened for zoonotic pathogens, for a total of 39 species (i.e., only 33% of the Asian species). The distribution of Asian primate species (34) screened across genera is as follows: *Macaca* sp. (*N* = 16 species among 22), *Pongo* sp. (*N* = 2 species among 3), *Presbytis* sp. (*N* = 5 species among 17), *Semnopithecus* sp. (*N* = 3 species among 8), *Trachypithecus* sp. (*N* = 5 species among 20), *Nasalis* sp. (*N* = 1 species among 1), *Nycticebus* sp. (*N* = 1 species among 8), *Cephalopachus* sp. (*N* = 1 species of 1), *Rhinopithecus* sp. (*N* = 1 species among 5), *Tarsius* sp. (*N* = 1 species among 13 species), and *Hylobates* sp. (*N* = 2 species among 9). The genus *Macaca* has been the most studied primate genus, covering about 75% of the included studies (Figure 3). Among the macaque species, more than half of the studies (65%) were carried out on *M. fascicularis* and *M. mulatta*.

#### 3.1.3 Diagnostic modalities

**Figure 4:**
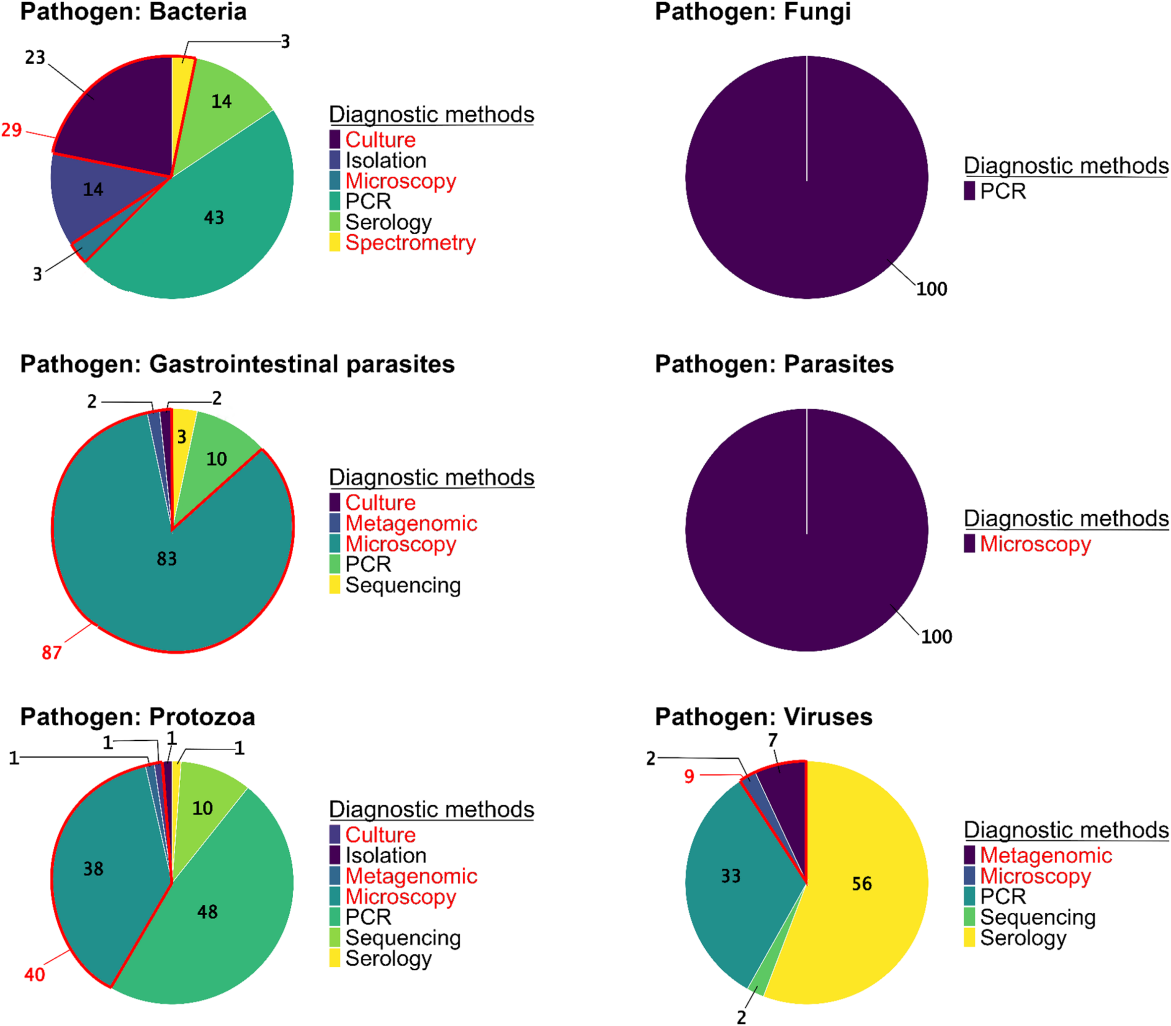
The proportions of pathogen detection methods used in the studies per pathogen group (*N* = 223 studies). Diagnostic methods are specified as generic (red) or specific (black).

Regarding the methods used to detect pathogens, PCR (41% of studies, *N* = 73 studies) and microscopy (32%, *N* = 56) were the most frequent, followed by serology (16%, *N* = 28). Among studies using microscopy to identify pathogens, different techniques were employed including (a) direct optic examination with staining (36%, *N* = 20), (b) direct optic examination without staining (61%, *N* = 34), and (c) direct electron microscopy examination, which was used in a few studies (4%, *N* = 2). Conversely, other detection methods such as bacteriological culture/isolation, spectrometry, and metagenomics were rare (11%, *N* = 20, including five studies using metagenomics). Generic detection methods were predominantly used in the detection of GI parasites (87%, *N* = 52) and blood-borne parasites (100%, *N* = 1). Conversely, specific detection methods were primarily employed for protozoa (60%, *N* = 50), fungi (100%, *N* = 3), bacteria (66%, *N* = 11), and viruses (91%, *N* = 39) (Figure 4). When examining the methods used to detect each group of pathogen, the results show a predominance of PCR in detection of fungi (100%, *N* = 3), bacteria (41%, *N* = 13), and protozoa (48%, *N* = 40). Viral infections were primarily detected indirectly through serology (56%, *N* = 24) or directly by PCR (33%, *N* = 14). Microscopy, a generic method, remains essential for the identification of GI parasites (83%, *N* = 50). Among these studies, 67% used direct optic examination without staining, 31% used direct optic examination with staining, and only 2% used direct electron microscopy examination. In microscopy-based studies on protozoa (38%, *N* = 32), both direct optic examination with and without staining were equally utilized. By contrast, advanced generic methods such as metagenomics were used in very few cases, with only 2% of studies on GI parasites (*N* = 1 study), 1% of studies on protozoa (*N* = 1), and 7% of studies on viruses (*N* = 3) (Figure 4).

#### 3.1.4 Groups of infectious agents and transmission pathways

**Figure 5:**
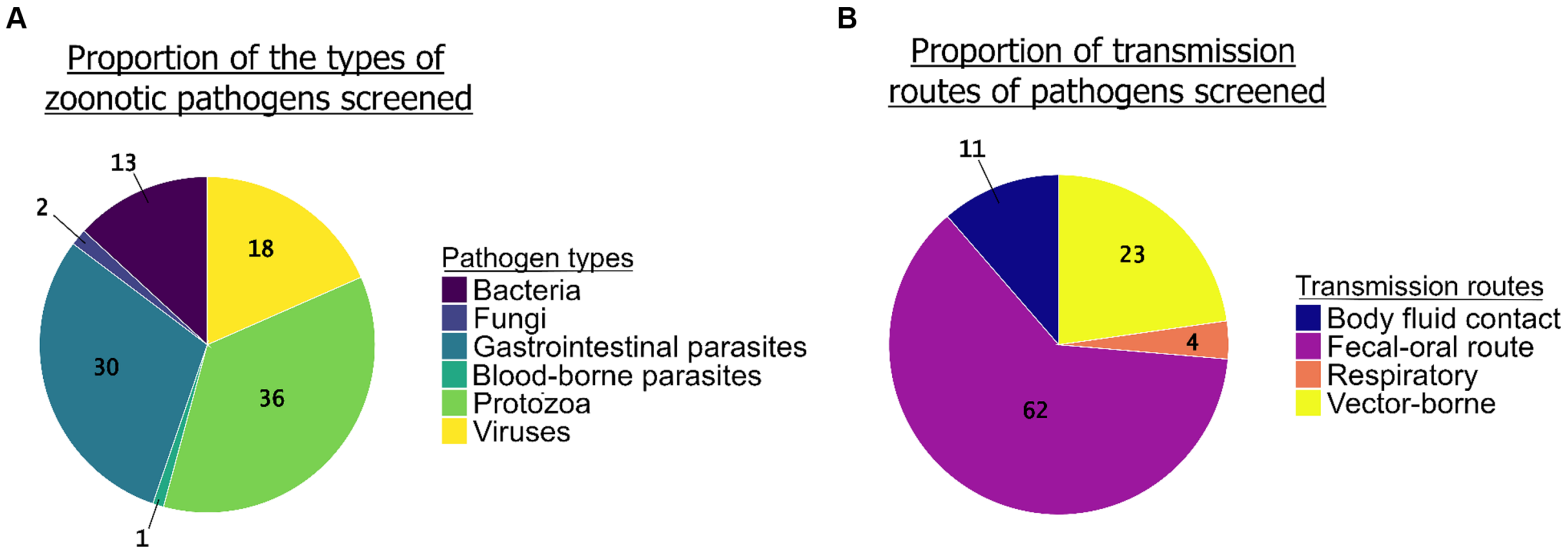
The proportion of groups of zoonotic pathogens screened (*N* = 190 studies) (**A**, left graph) and the proportion of their transmission routes (**B**, right graph) (*N* = 167 studies).

The review enabled us to highlight unequal sampling efforts between the groups of pathogenic agents screened (Figure 5A). Protozoa (*N* = 68 studies) and GI parasites (*N* = 57 studies) together represented more than two thirds of the pathogens screened in the studies (36 and 30%, respectively), while bacteria (*N* = 23 studies) and viruses (*N* = 35 studies) were less studied (12 and 19%, respectively). We found only three studies that screened for fungi and one study for blood-borne parasites. Regarding the transmission pathways, the most common route of transmission of the zoonotic pathogens screened in the studies was the fecal-oral route (62%, *N* = 104) followed by the vector-borne route (23%, *N* = 38), body fluid contact (11%, *N* = 19), and the respiratory route (4%, *N* = 6) (Figure 5B).

Gastrointestinal parasites and protozoa were found in the largest number of primate genus (in *N* = 10 and *N* = 8 genera, respectively). Conversely, fungi, blood-borne parasites, and bacteria were screened and reported in very few primate genera (*N* = 1, 1, and 2, respectively). For example, *Nycticebus* sp., *Cephalopachus* sp., and *Tarsius* sp. have only been studied for GI parasites, with no other pathogen groups reported. In contrast, *Macaca* sp. has been extensively studied on various groups of pathogens: protozoa (37%, *N* = 58 studies), GI parasites (26%, *N* = 40), viruses (19%, *N* = 30), bacteria (15%, *N* = 24), and fungi (2%, *N* = 3). Regarding the diagnostic methods used for pathogen detection, the results show a predominance of microscopy and PCR, although this varies by primate genus. For example, microscopy was the sole method used in studies on *Hylobates* sp., *Tarsius* sp., *Nycticebus* sp., and *Cephalopachus* sp. Microscopy was also the most commonly method used with *Nasalis* sp. (71%, *N* = 5 studies), *Pongo* sp. (36%, *N* = 4 studies), *Presbytis* sp. (60%, *N* = 3 studies), and *Trachypithecus* sp. (56%, *N* = 5 studies). Finally, PCR was also prevalent in studies on *Macaca* sp. (39%, *N* = 65 studies) and *Rhinopithecus* sp. (67%, *N* = 4 studies) (Table 1).

**Table 1:** Percentage (and number) of studies relative to each zoonotic pathogen group, and each diagnostic method, per Asian primate genus.^*^

Primate genus	Pathogen group % (no.)						Diagnostic method % (no.)							
	PGI	Protozoa	Viruses	Bacteria	Fungi	Blood-borne parasites	Culture	Metagenomic	Microscopy (a, b, c)	Spectrometry	Isolation	Serology	PCR	Sequencing
*Macaca sp.*	26 (40)	37 (58)	19 (30)	15 (24)	2 (3)	0	7 (11)	2 (3)	27 (44)	1 (1)	4 (6)	15 (25)	39 (65)	6 (10)
*Presbytis sp.*	44 (4)	33 (3)	11 (1)	0	0	11 (1)	0	20 (1)	60 (3)	0	20 (1)	0	0	0
*Trachypithecus sp.*	60 (6)	20 (2)	10 (1)	10 (1)	0	0	0	0	56 (5)	0	11 (1)	0	22 (2)	11 (1)
*Semnopithecus sp.*	43 (3)	29 (2)	29 (2)	0	0	0	0	0	57 (4)	0	0	0	43 (3)	0
*Pongo sp.*	50 (7)	36 (5)	14 (2)	0	0	0	9 (1)	0	36 (4)	0	0	18 (2)	27 (3)	9 (1)
*Rhinopithecus sp.*	0	50 (2)	50 (2)	0	0	0	0	0	0	0	0	17 (1)	67 (4)	17 (1)
*Nasalis sp.*	86 (6)	14 (1)	0	0	0	0	0	0	71 (5)	0	0	0	14 (1)	14 (1)
*Hylobates sp.*	50 (1)	50 (1)	0	0	0	0	0	0	100 (2)	0	0	0	0	0
*Tarsius sp.*	100 (1)	0	0	0	0	0	0	0	100 (1)	0	0	0	0	0
*Nycticebus sp.*	100 (1)	0	0	0	0	0	0	0	100 (1)	0	0	0	0	0
*Cephalopachus sp*	100 (1)	0	0	0	0	0	0	0	100 (1)	0	0	0	0	0

Diagnostic methods are specified as generic (blue) or specific (red). Microscopy diagnostic methods include: (a) direct optic examination with staining, (b) direct optic examination without staining (only flotation and/or sedimentation), and (c) direct electron microscopy examination. Asian primate genera for which no studies have been found on zoonotic pathogens include *Pygathrix* sp., *Hoolock* sp., *Nomascus* sp., *Symphalangus* sp., *Simias* sp., *Loris* sp. and *Carlito* sp.

### 3.2 Inventory of zoonotic pathogens

#### 3.2.1 Protozoa

Protozoa were the most studied zoonotic agents reported (*N* = 68 studies). Forty-two species of protozoa were identified, including 35 species transmitted by the fecal-oral route, which was the most common route (*N* = 47 studies), and seven species transmitted by the vector-borne route (*N* = 20 studies) (Supplementary Table S1). Among the vector-borne protozoa, the most common diagnostic method was PCR (*N* = 18 studies). The two genera of vector-borne protozoa reported were *Hepatocystis* sp. (*N* = 2 studies) and *Plasmodium* sp. (*N* = 18 studies). Studies mainly reported species belonging to the genus *Plasmodium* (*N* = 6 species). More specifically, *Plasmodium cynomolgi* and *Plasmodium inui* were detected in the largest number of host primate species, including *Macaca fascicularis*, *M. nemestrina*, *M. leonina*, *M. arctoides*, *M. sinica* (only for *P. cynomolgi*), *M. radiata*, and *Presbytis entellus* (only for *P. cynomolgi*) (53–66). In addition, *Plasmodium falciparum* was detected in *M. radiata* and *M. mulatta* (56), while *P. knowlesi* was detected in *M. fascicularis*, *M. nemestrina*, and *M. arctoides* (53, 54, 57, 59–61, 64–69). These two zoonotic *Plasmodium* species are known to cause severe cases of malaria in humans. In fact, *Plasmodium falciparum* is responsible for the most severe and deadly forms of malaria, with complications such as severe anemia, coma, and multi-organ failure (70). *Plasmodium knowlesi*, which has recently been recognized as a human pathogen, can also cause severe clinical symptoms including respiratory and renal failure. However, most cases respond well to prompt treatment (71).

#### 3.2.2 GI parasites

Helminthic GI parasites were the second most studied pathogens (*N* = 57 studies), with a total of 63 species that have been reported in all habitat types. Nematodes were the most detected helminthic GI parasites: indeed, of the 63 species described, 42 were nematodes (Supplementary Table S2). Certain species of nematodes were found in many host primates and reported in several studies. This is the case for *Strongyloides* sp., detected in 19 primate species in 33 studies; *Trichostrongylus* sp., detected in 17 primate species in 23 studies; and *Trichuris* sp., reported in 19 primate species in 32 studies (24, 25, 72–112). Concerning the other species of helminthic GI parasites, eight species of cestodes (i.e., *Diphyllobothrium* sp., *Dipylidium caninum*, *Echinococcus* sp., *Hymenolepsis* sp., *Hymenolepsis diminuta*, *Hymenolepsis nana*, *Moniezia* sp., and *Taenia* sp.) have been reported (24, 25, 85, 86, 91, 92, 95, 97, 99, 100, 102, 106, 108, 109, 113–115) (Supplementary Table S3), and a total of 13 species of trematodes were detected in all different habitat types (Supplementary Table S4).

#### 3.2.3 Viruses

In total, 45 species of zoonotic viruses were reported in Asian primates of the included studies (Supplementary Table S5). Viruses represented the third most studied type of zoonotic pathogens (*N* = 35 studies). The majority of viruses found in urban habitats are transmitted by body fluid contact (*N* = 9 viruses) (116–123). Regarding diagnostic modalities, most studies on viruses used serological methods (*N* = 24 studies) or PCR (*N* = 14 studies). Simian foamy virus (116–120, 123), Japanese encephalitis virus, and dengue virus (74, 124–130) were the most studied viruses (*N* = 6, *N* = 5, and *N* = 5 studies, respectively). However, dengue and chikungunya were the viruses reported in the largest number of primate species (five primate species for each virus).

#### 3.2.4 Bacteria, fungi, and blood-borne parasites

We found few studies screening for bacteria, fungi, and blood-borne parasites (*N* = 23 studies, *N* = 3 and *N* = 1, respectively). Regarding zoonotic fungi, only *Enterocytozoon bieneusi* was detected by PCR in *M. mulatta and M. assamensis* (131–133). Two genera of blood-borne pathogens *Brugia* sp. and *Wuchereria* sp. were found by microscopic analyses in *Presbytis cristatus* (112). For bacteria, a total of 30 species were reported, but in only three primate genera, i.e., *Pongo* sp. (*N* = 1 study), *Trachypithecus* sp. (*N* = 1 study), and *Macaca* sp. (*N* = 24 studies) (Supplementary Table S6). The bacterium *Escherichia coli* found in *M. mulatta, M. fascicularis*, and *M. fuscata* (38, 88, 134–136), along with *Staphylococcus aureus* found in *M. mulatta*, *M. fascicularis*, and *Trachypithecus cristatus* (80, 137–140) were the most common bacteria (*N* = 5 and *N* = 5 studies, respectively). Apart from the typical oral-fecal route, there were two bacterial species transmitted via the vector-borne route—*Bartonella quintana*, a zoonotic bacterium that causes fever in humans and was found in the Japanese macaque (*M. fuscata*) and *M. fascicularis* (141, 142), and *Candidatus Mycoplasma haemomacaque* in *M. fascicularis* (143)—and two bacterial species transmitted by the respiratory route, namely *Mycobacterium tuberculosis* in *M. mulatta* and *M. fascicularis* (144) *and Streptococcus* sp. in *M. fascicularis* (145–147).

### 3.3 Comparison of pathogen specific richness between habitat types

**Figure 6:**
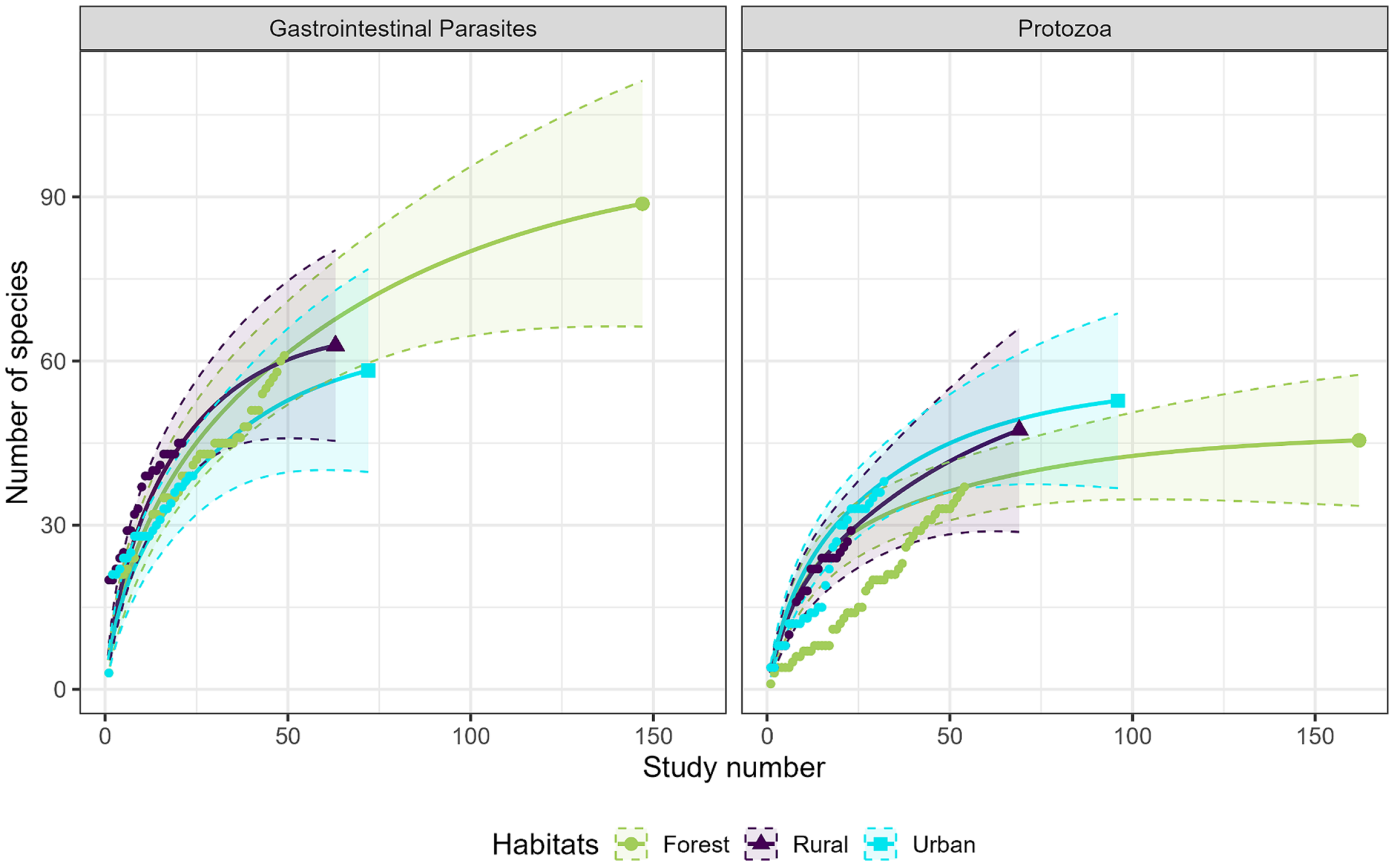
Comparison of the species accumulation curves (dotted line) and the extrapolated species rarefaction curves (solid line with confidence intervals) between urban, rural, and forest habitats for gastrointestinal parasites (left graph) and protozoa (right graph).

**Figure 7:**
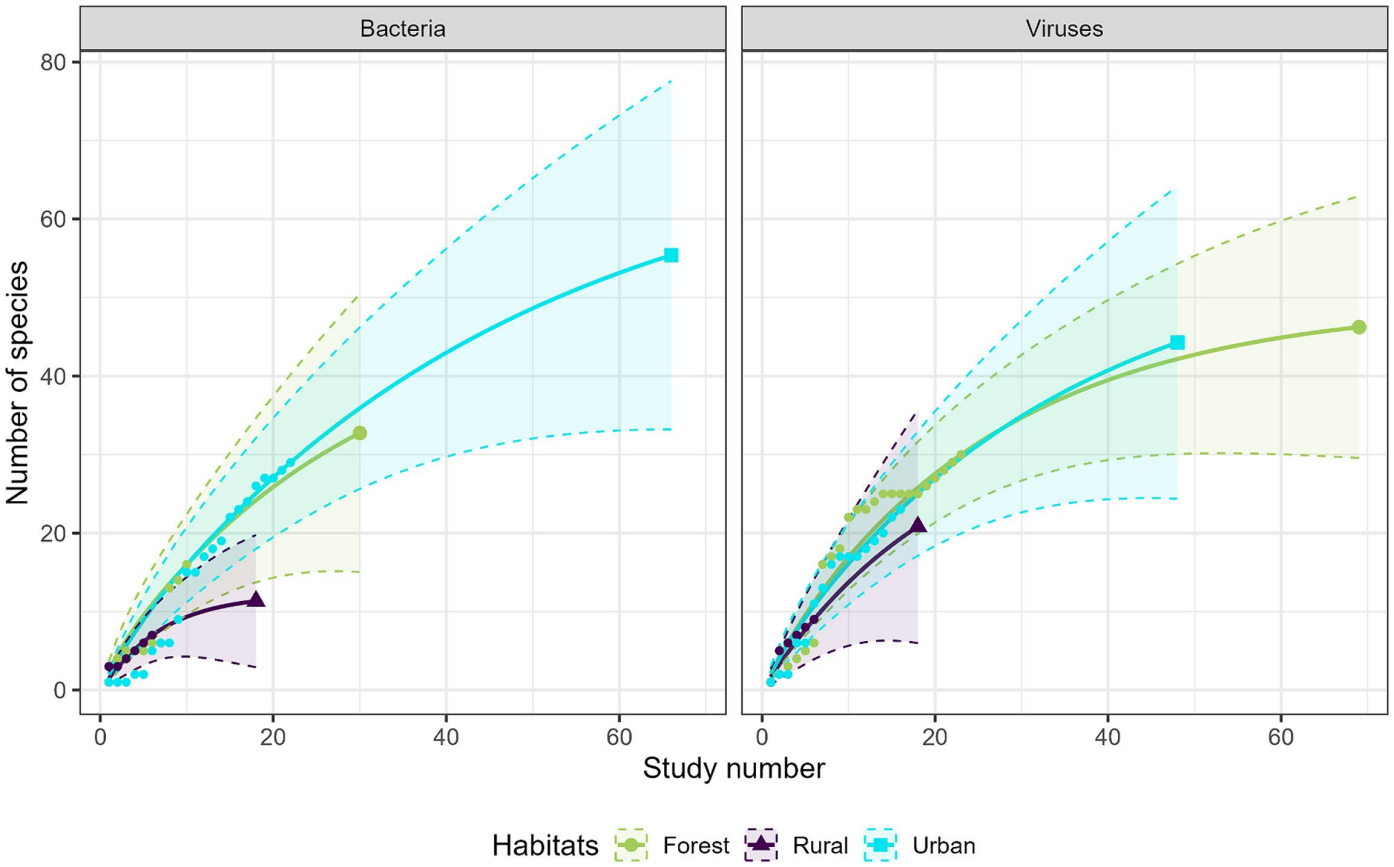
Comparison of the species accumulation curves (dotted line) and the extrapolated species rarefaction curves (solid line with confidence intervals) between urban, rural, and forest habitats for bacteria (left graph) and viruses (right graph).

Most studies were conducted in forest habitats (*N* = 107 studies), followed by urban habitats (*N* = 75 studies) and rural habitats (*N* = 43 studies). From a descriptive perspective, comparison of the accumulation curves and the associated rarefaction curves highlighted a lower species accumulation trend in urban habitats for GI parasites, and in forest habitats for protozoa (Figure 6). A higher exhaustive species richness of GI parasites was found in forest habitat compared with the other two habitats. We also found a reduced protozoan richness in forest habitat and the highest richness in urban habitat (Figure 6). Regarding bacteria, species richness was the highest in urban habitat and the lowest in rural habitat (Figure 7). Finally, viruses were unfrequently reported in rural habitat (Figure 7). Despite these trends, the confidence intervals of the rarefaction curves showed broad overlap among the habitat types for all pathogen groups, which suggest no statistical difference in the predominance of zoonotic species between urban, rural, and forest habitats. Additionally, all pathogen groups (except Protozoa in forest habitat) displayed non-asymptotic accumulation and rarefaction curves (Figures 6, 7). This indicates that the saturation level (i.e., exhaustivity) for pathogen taxa was not reached in any habitat type, supporting that the sampling effort was insufficient to extrapolate differences, and calling for further surveillance to identify more pathogens.

## 4 Discussion

The COVID-19 pandemic has been a reminder of the paramount importance of zoonotic diseases for global health (148). Many zoonoses originate from or have as reservoirs non-human primates (30, 149). Asia has been the origin of several suspected zoonotic transmission events over the past decades (e.g., previous SARS outbreaks in the 2000s, Nipah virus in 1998, and H5N1 avian influenza virus in the 2000s); underscoring the critical role of the region in the transmission dynamics and the emergence of zoonotic diseases (150). Surveillance of wildlife hosts and potential reservoirs is a crucial initial step to mitigate the risk of future pandemics (13). Given their genetic similarity to humans, non-human primates are important potential reservoirs of zoonotic infections (151). To limit the risk of transmission, it is essential to identify and document the zoonotic pathogens carried by free-ranging primates that often lead to interact with humans. In this review, we conducted the first comprehensive inventory of the various groups of zoonotic pathogens identified in non-captive Asian primates in urban, rural, and forest habitats. By doing so, we have highlighted current research gaps regarding zoonotic pathogens in wild Asian primates, focusing on coverage in primate taxonomic hosts, pathogen groups, and diagnostic methods. As an example, we could not draw a robust conclusion about the potential differences in pathogen-specific richness across habitat types due to insufficient research efforts.

Our review included 152 studies on zoonotic pathogens encompassing reports on 39 primate species from 15 Asian countries. This sampling represents only 33% of the extant primate species found in Asia (34). Hence, a small number of species, especially those of the *Macaca* genus, are oversampled in infectious disease studies, while many others are disregarded such as the doucs (*Pygathrix* sp.) or some gibbons (*Hoolock* sp. and *Nomascus* sp.). This result confirms a significant taxonomic bias of sampling in the scientific literature that has been raised previously. In their 2007 review, Hopkins and Nunn (45) examined research on infectious agents in primates throughout the world, and found that African primates were sampled twice as much as Asian primates. The disparities we found across Asian primate taxa could be explained by several factors. Sampling in infectious disease research is influenced by the geographic range and the locomotion mode of the primate species (152). Widespread and semi-terrestrial species are sampled more frequently than geographically restricted and strictly arboreal species. Consistently, the overrepresentation of *M. fascicularis* and *M. mulatta* may be ascribed to their extensive distribution range across Asia compared with other primate species (153). Moreover, *M. fascicularis* and *M. mulatta* are conspicuous and often found in anthropogenic habitats where they are more terrestrial, making their access and sample collection easier compared with elusive species in remote areas (154). Interestingly, while *Semnopithecus entellus*, a terrestrial species that often inhabits human-modified environments (2, 155) would represent an easy and relevant candidate for sampling and assessing zoonotic risks, it was underrepresented in our dataset, comprising only 2% (*N* = 5 studies) of the studies. Conversely, survey effort has been greater for emblematic and threatened species such as orangutans (*Pongo* sp.) (*N* = 10 studies, 5%). Furthermore, since this review primarily examined English-language publications, it could be advisable for future bibliographic searches to encompass literature published in Oriental languages, with careful consideration of potential publication biases.

The under-sampling of many primate taxa in surveillance studies may pose a public health risk as these primates are potential unknown reservoirs of zoonotic pathogens. This risk is exacerbated by the context of the growing demand for bushmeat and wildlife products, which is also observed in Asia (156, 157). The growing threat of illegal hunting has implications for primate conservation and human health as it intensifies the potential for the circulation of zoonotic pathogens (158, 159). As evidenced by several examples in Africa, the manipulation and consumption of primate meat facilitates the transmission of zoonotic diseases to humans, resulting in dire consequences, such as the emergence of HIV or Ebola virus outbreaks (30, 149). In Asia, although lorises (*Loris* sp. and *Nycticebus* sp.) and tarsiers (*Tarsius* sp., *Cephalopachus* sp., and *Carlito* sp.) are widely traded as pets, presenting risks of zoonotic transmission (160, 161), there are still very few infectious disease studies on these species, with the exception of a small fraction screening for GI parasites.

Another publication bias underscored by this review concerns the uneven allocation of sampling efforts regarding the types of screened pathogenic agents and studied transmission routes. Overall, we found that the focus of most empirical studies that examined zoonotic pathogens in free-ranging Asian primates was on protozoa and GI parasites, with most of the identified agents transmitted through the fecal-oral route. Concerning GI parasites, nematodes such as *Strongyloides* sp., *Trichostrongylus* sp., and *Trichuris* sp. were the most reported infectious agents. Viruses, bacteria, blood-borne parasites and fungi have been documented less frequently in the literature on Asian primates. These results are consistent with Hopkins and Nunn (45) and Cooper and Nunn (152) studies, who showed that helminths are the most commonly studied pathogens in primates, while bacteria, viruses, and fungi are the least investigated infectious agents. However, it is important to note that most GI parasites have frequent asymptomatic carriage in wildlife, which may not always reflect a significant health risk for animals or humans (162, 163). The oversampling of pathogens transmitted by the fecal-oral route, such as *Strongyloides* sp. and *Entamoebas* sp., could be explained by logistic and ethical constraints related to the sample matrix necessary for diagnosis. Indeed, fecal samples collected noninvasively from the ground are an easy and conventional tool for evaluating zoonotic pathogens in primates, in particular GI parasites (164). Given the vulnerable status of many primate species and ethical restrictions, it may be difficult to obtain authorizations to collect blood or other body fluid samples in the wild (165). Therefore, although molecular techniques with fecal samples can be used to identify diverse types of agents such as blood-borne pathogens (166), microscopy allowing researchers to identify macroparasites and protozoa are commonly used in the field. In future studies, it would be beneficial to expand surveillance strategies through other types of non-invasively collected sample matrices such as saliva, hairs or urine, which can also be gathered without harming the animals (167). These alternative samples could provide valuable insights into a broader range of pathogens, including viruses and bacteria or those difficult to detect through fecal sample analysis, thus enhancing our understanding and management of zoonotic diseases.

So far, PCR, microscopy, and serology have been the most prevalent methods used in studies on Asian primate infections. For all pathogens, except GI parasites and blood-borne parasites, more than half of the studies used pathogen-specific detection methods requiring an *a priori* selection of the pathogens potentially present in the population. Even though microscopy is a generic detection method (that is sometimes supplemented by more specific detection methods such as PCRs to allow the identification beyond the genus level), it is mainly used for the detection of GI parasites and protozoa. The predominance of those pathogen-specific methods likely skewed the true representation of the infectious agents. Indeed, while sensitive, specific, and efficient methods such as real-time polymerase chain reaction (qPCR) are routinely used for known pathogens, the identification of emerging or unknown pathogens is more challenging (168). In this regard, the *de novo* metagenomics approach has proved to be a powerful new tool with infinite fields of application (169). For example, many novel and divergent viruses can be detected simultaneously and genetically characterized for the first time (170). In addition, metagenomic analyses of the microbial community also provide important insights and tools to monitor the health and nutritional status of primates and thus contribute to primate conservation (171). Generic next-generation sequencing approaches, through a wide variety of samples (i.e., feces, blood, nasal swab, saliva, and biological tissues), are likely to shed light on little known or novel zoonotic pathogens in primates (169) such as the ChiSCVs virus detected in stool samples of wild chimpanzees (172) or the Primate Bocaparvovirus Species 3 discovered in wild rhesus macaques (173). However, the relatively limited use of generic methods can likely be attributed to the high cost associated with next-generation sequencing techniques. Primate conservation research in developing countries is often a low priority (174), given the growth needs of local populations and the lack of technical resources and funding (175). Despite improved efforts in recent years, there remains a lack of international collaboration, which reduces opportunities for local research, capacity building, and access to cutting-edge technologies needed to improve the detection of zoonotic EIDs and the underpinning mechanisms (175, 176).

Based on the existing literature, we were unable to confidently determine whether the type of habitat influences the diversity of zoonotic agents that infect wild Asian primates. With respect to protozoa, bacteria, viruses, and GI parasites, even though their specific richness did not show significant variations across forest, rural, and urban habitats, the inadequacy of sampling effort is apparent from the absence of asymptotes in the rarefaction curves. Yet, anthropogenic disturbances such as forest degradation and land-use conversion are suspected to deeply interact with infectious diseases in primates, notably through a multiplication of direct and indirect contacts with humans and domestic animals (30). In rhesus macaques, habitat attributes correlated with host density and appeared to be a significant determinant of GI parasite infections. Parasitic richness was higher in large macaque groups interacting with human communities and livestock in (peri-)urban habitats, although parasitic prevalence was higher in rural habitats (95). Consistently, the prevalence of *Salmonella* sp. and *E. coli* was higher in provisioned groups of rhesus macaques interacting with humans in anthropogenic habitats (38). Conversely, another study in Indonesia demonstrated that anthropogenic landscape components decreased the prevalence and intensity of GI parasites in long-tailed macaques, probably due to good nutritional conditions following heavy food provisioning near human settlements (25). The prevalence and risk of transmission of viruses transmitted through physical contact or aerosols are expected to be higher in urban habitats such as at touristic sites and temples in Asia (116, 118, 119), where close and frequent human–primate contacts are common (101). In rural landscapes, agricultural practices, such as the use of antibiotics, can also contribute to drug resistance of bacteria and therefore increase their prevalence in primates (135). Conversely, other several studies on African primates have shown a higher richness and prevalence of GI parasites in populations from disturbed forests compared with more preserved habitats (177, 178). Finally, the prevalence of vector-borne pathogens, such as protozoa responsible for malaria, may be increased by forest degradation and associated changes in vector (anopheline mosquitoes) and host (*Macaca* sp.) density (179, 180).

Nevertheless, it is important to acknowledge that the descriptive categorization of habitats based on site descriptions we used in this review to delineate three categories (i.e., urban, rural, and forest), may entail certain limitations in the results. A more empirical approach using satellite images of land cover could potentially provide a more accurate representation of the environmental complexity by considering finer variations in land use and thereby capturing a broader spectrum of anthropogenic influences on primate habitats. In sum, the influences of anthropogenic components on primate infections appear complex. Urgent additional comparative studies are needed to investigate changes in primate-pathogen dynamics in rapidly changing environments, particularly among primate populations inhabiting habitats with varying degrees of human disturbances (30, 40).

## 5 Conclusion

The different biases highlighted in this literature review warrant further investigation, particularly on the under-screened primate species and on a wider range of etiological agents by using generic diagnostic methods. Primates are good candidates as sentinels for the surveillance of zoonotic diseases, particularly in Asia, where their close spatial proximity to humans is rapidly increasing. This endeavor requires researchers to address knowledge gaps regarding the risks and mechanisms associated with zoonotic transmissions. For example, it would be promising to improve our understanding of the behavior and socio-ecology of synanthropic primates. So far, few studies have focused on the risk factors of disease transmission associated with primate social dynamics, personality traits, and risk-taking behaviors promoting contacts with humans, domestic animals, or shared resources (40). It is worth emphasizing that such knowledge into primate health and the mechanisms of disease transmission also has substantial implications for primate conservation (171).

A One Health conceptual approach grounded in multidisciplinary collaborations is crucial for conducting action research on the emergence and transmission of zoonoses (181, 182). Establishing effective preventive measures requires a targeted surveillance of potential zoonotic reservoirs to identify mechanisms and risk factors of EIDs, and to raise awareness among populations about zoonotic risks. Today, considering previous sanitary crises associated with wildlife reservoirs [e.g., Ebola, Middle East respiratory syndrome (MERS), and COVID-19], it is essential to draw on the lessons that have been learned to make informed decisions. Prioritizing preventive measures, such as identifying infection reservoirs, implementing surveillance, and communicating risks, is advised over reactive measures like implementing physical barriers and restricting human populations in response to zoonotic outbreaks.

## Data availability statement

The original contributions presented in the study are included in the article/Supplementary material, further inquiries can be directed to the corresponding author.

## Author contributions

LP: Conceptualization, Data curation, Formal analysis, Funding acquisition, Investigation, Methodology, Resources, Software, Visualization, Writing – original draft. AH: Formal analysis, Methodology, Software, Supervision, Validation, Writing – review & editing. SA: Validation, Writing – review & editing. MG: Conceptualization, Funding acquisition, Methodology, Project administration, Supervision, Validation, Writing – review & editing. FB: Conceptualization, Funding acquisition, Methodology, Project administration, Supervision, Validation, Writing – review & editing.
