# Supplementary material for: Zoonotic pathogens in wild Asian primates: a systematic review highlighting research gaps
Source: Front Vet Sci. 2024 Jun 27;11:1386180. doi: 10.3389/fvets.2024.1386180 (PMC11238137; doi:10.3389/fvets.2024.1386180)
Supplement: Supplementary file 1 [file Data_Sheet_1.docx]

Supplementary Material S7

Zoonotic pathogens in wild Asian primates: A systematic review highlighting research gaps

Laurie Patouillat^1,2*^, Alain Hambuckers^1^, Sena Adi Subrata^3^, Mutien-Marie Garigliany^2^†, Fany Brotcorne^1^†

*** Correspondence:** Laurie Patouillat ; laurie.patouillat@uliege.be

# Supplementary References Cited in the Inventory Presented in Supplementary Materials S1 to S6

182. Yang J, Okyere SK, Zheng J, Cao B, Hu, Y. Seasonal prevalence of gastrointestinal parasites in macaques (Macaca thibetana) at Mount Emei Scenic Area in China. Animals. (2022) 12(14):1816. doi: 10.3390/ani12141816

183. Yoshikawa H, Wu Z, Pandey K, Pandey BD, Sherchand JB, Yanagi T, Kanbara H. Molecular characterization of Blastocystis isolates from children and rhesus monkeys in Kathmandu, Nepal. Vet Parasitol. (2009) 160(3–4):295–300. doi: 10.1016/j.vetpar.2008.11.029

184. Junaidi J, Cahyaningsih U, Purnawarman T, Latif H, Sudarnika E, Farida F, Maryatun M. The distribution of intestinal amoebae in wild long-tailed macaques (Macaca fascicularis) in Sabang City, Aceh Province, Indonesia. Trends Sci. (2022) 19(1):1717. doi: 10.48048/tis.2022.1717

185. Yu M, Yao Y, Xiao H, Xie M, Xiong Y, Yang S, Ni Q, Zhang M, Xu H. Extensive prevalence and significant genetic differentiation of Blastocystis in high- and low-altitude populations of wild rhesus macaques in China. Parasites Vectors. (2023) 16(1):107. doi: 10.1186/s13071-023-05691-7

186. Debenham JJ, Tysnes K, Khunger S, Robertson LJ. Occurrence of Giardia, Cryptosporidium, and Entamoeba in wild rhesus macaques (Macaca mulatta) living in urban and semi-rural North-West India. Int J Parasitol Parasites Wildl. (2017) 6(1):29–34. doi: 10.1016/j.ijppaw.2016.12.002

187. Sricharern W, Inpankaew T, Keawmongkol S, Supanam J, Stich RW, Jittapalapong S. Molecular detection and prevalence of Giardia duodenalis and Cryptosporidium spp. among long-tailed macaques (Macaca fascicularis) in Thailand. Infect Genet Evol. (2016) 40:310–314. doi: 10.1016/j.meegid.2016.02.004

188. Jia R, Wen X, Guo Y, Xiao L, Feng Y, Li N. Decline in Cryptosporidium infection in free-ranging rhesus monkeys in a park after public health interventions. Front Cell Infect Microbiol. (2022) 12:901766. doi: 10.3389/fcimb.2022.901766

189. Chang AM, Chen CC, Huffman MA. Entamoeba spp. in wild formosan rock macaques (Macaca cyclopis) in an area with frequent human-macaque contact. J Wildl Dis. (2019) 55(3):608–618. doi: 10.7589/2018-04-113

190. Chrismanto D, Lastuti ND, Suwanti L, Hastutiek P, Kurniawati D, Paramita C, Witaningrum A, Pratiwi A. Diversity of Entamoeba spp. in long-tailed macaque (Macaca fascicularis) in Baluran and Alas Purwo National Parks, Indonesia. Biodiversitas. (2022) 3(9):4524–4531. doi: 10.13057/biodiv/d230919

191. Stuart P, Yalcindag E, Ali IKM, Pecková R, Nurcahyo W, Morrogh-Bernard H, Foitová I. Entamoeba histolytica infections in wild and semi-wild orangutans in Sumatra and Kalimantan. Am J Primatol. (2020) 82(5):e23124. doi: 10.1002/ajp.23124

192. MacIntosh AJ, Hernandez AD, Huffman MA. Host age, sex, and reproductive seasonality affect nematode parasitism in wild japanese macaques. Primates. (2010) 51(4):353–364. doi: 10.1007/s10329-010-0211-9

193. Arizono N, Yamada M, Tegoshi T, Onishi K. Molecular identification of Oesophagostomum and Trichuris eggs isolated from wild Japanese macaques. Korean J Parasitol. (2012) 50(3):253–257. doi: 10.3347/kjp.2012.50.3.253

194. Labes EM, Nurcahyo W, Deplazes P, Mathis A. Genetic characterization of Strongyloides spp. from captive, semi-captive and wild bornean orangutans (Pongo pygmaeus) in Central and East Kalimantan, Borneo, Indonesia. Parasitology. (2011) 138(11):1417–1422. doi: 10.1017/S0031182011001284

195. Cardeti G, Cersini A, Manna G, De Santis P, Scicluna MT, Albani A, Simula M, Sittinieri S, De Santis L, De Liberato C, Ngakan PO, Wahid I, Carosi M. Detection of viruses from feces of wild endangered Macaca maura: a potential threat to moor macaque survival and for zoonotic infection. BMC Vet Res. (2022) 18(1):418. doi: 10.1186/s12917-022-03506-y

196. Jones-Engel L, Engel GA, Schillaci MA, Babo R, Froehlich J. Detection of antibodies to selected human pathogens among wild and pet macaques (Macaca tonkeana) in Sulawesi, Indonesia. Am J Primatol. (2001) 54:171–178. doi: 10.1002/ajp.1021

197. Ortiz-Cam L, Jones-Engel L, Mendoza P, Castillo-Neyra R. Association between seroprevalence of Measles virus in monkeys and degree of human-monkey contact in Bangladesh. One Health. (2023) 17:100571. doi: 10.1016/j.onehlt.2023.100571

198. Oberste MS, Feeroz MM, Maher K, Nix WA, Engel GA, Hasan KM, Begum S, Oh G, Chowdhury AH, Pallansch MA, Jones-Engel L. Characterizing the Picornavirus landscape among synanthropic nonhuman primates in Bangladesh, 2007 to 2008. J Virol. (2012) 87(1):558–571. doi: 10.1128/jvi.00837-12

199. Burke DS, Heisey GB. Wild malaysian cynomolgus monkeys are exposed to hepatitis A virus. Am J Trop Med Hyg. (1984) 33(5):940–944. doi: 10.4269/ajtmh.1984.33.940

200. Pavri K. Clinical, clinicopathologic, and hematologic features of Kyasanur Forest disease. Rev Infect Dis. (1989) 11(Ii):854–859. doi: 10.1093/clinids/11.Supplement_4.S854

201. Mourya DT, Yadav PD, Sandhya VK, Reddy S. Spread of Kyasanur Forest disease, Bandipur Tiger Reserve, India, 2012-2013. Emerg Infect Dis. (2013) 19(9):1540–1541. doi: 10.3201/eid1909.121884

202. Ain-Najwa MY, Yasmin AR, Arshad SS, Omar AR, Abu J, Kumar K, Mohammed HO, Natasha JA, Mohammed MN, Bande F, Abdullah ML, Rovie-Ryan J. Exposure to zoonotic West Nile virus in long-tailed macaques and bats in peninsular Malaysia. Animals. (2020) 10(12):2367. doi: 10.3390/ani10122367

203. Saechan V, Tongthainan D, Fungfuang W, Tulayakul P, Ieamsaard G, Ngasaman R. Natural infection of leptospirosis and melioidosis in long-tailed macaques (Macaca fascicularis) in Thailand. J Vet Med Sci. (2022) 84(5):700–706. doi: 10.1292/jvms.21-0514

204. Tegner C, Sunil-Chandra NP, Wijesooriya WRPLI, Perera BV, Hansson I, Fahlman Å. Detection, identification, and antimicrobial susceptibility of Campylobacter spp. and Salmonella spp. from free-ranging nonhuman primates in Sri Lanka. J Wildl Dis. (2019) 55(4):879–884. doi: 10.7589/2018-08-199

205. Napit R, Manandhar P, Poudel A, Rajbhandari PG, Watson S, Shakya S, Pradhan SM, Sharma AN, Chaudhary A, Johnson CK, Mazet JK, Karmacharya D. Novel strains of Campylobacter cause diarrheal outbreak in rhesus macaques (Macaca mulatta) of Kathmandu Valley. PLoS One. (2023) 18:e0270778. doi: 10.1371/journal.pone.0270778

206. Rahman MK, Hassan MM, Islam S, Rostal MK, Uddin MH, Hagan E, Samad MA, Flora MS, Epstein JH, Islam A. Characterization and epidemiology of antimicrobial resistance patterns of Salmonella spp. and Staphylococcus spp. in free-ranging rhesus macaque (Macaca mulatta) at high-risk interfaces with people and livestock in Bangladesh. Front Vet Sci. (2023) 10:1103922. doi: 10.3389/fvets.2023.1103922

207. Brown R, Salgado-Lynn M, Jumail A, Jalius C, Chua TH, Vythilingam I, Ferguson HM. Exposure of primate reservoir hosts to mosquito vectors in malaysian Borneo. EcoHealth. (2022) 19(2):233-245. doi: 10.1007/s10393-022-01586-8

208. Gotoh S, Takenaka O, Watanabe K, Hamada Y, Kawamoto Y, Watanabe T, Suryobroto B, Sajuthi. Hematological values and parasite fauna in free-ranging Macaca hecki and the M.hecki/M.tonkeana hybrid group of Sulawesi Island, Indonesia. Primates. (2001) 42(1):27-34. doi: 10.1007/BF02640686

209. Mon HM, Feng M, Pattanawong U, Kosuwin R, Yanagi T, Kobayashi S, Putaporntip Chaturong, Jongwutiwes S, Cheng X, Tachibana H. Genotyping of Entamoeba nuttalli strains from the wild rhesus macaques of Myanmar and comparison with those from the wild rhesus macaques of Nepal and China. Infection, Genetics and Evolution. (2021) 92:104830. doi: 10.1016/j.meegid.2021.104830

210. Vaisusuk K, Saijuntha W, Sedlak S, Thanchomnang T, Pilap W, Suksavate W, Stensvold CR, Tantrawatpan C. Blastocystis subtypes detected in long-tailed macaques in Thailand—Further evidence of cryptic host specificity. Acta Tropica. (2018) 184:78-82. doi: 10.1016/j.actatropica.2017.09.002

211. Feng M, Yanagi T, Putaporntip C, Pattanawong U, Cheng X, Jongwutiwes S, Tachibana H. Correlation between genotypes and geographic distribution of Entamoeba nuttalli isolates from wild long-tailed macaques in Central Thailand. Infection, Genetics and Evolution. (2019) 70:114-122. doi: 10.1016/j.meegid.2019.02.030

212. Tachibana H, Yanagi T, Lama C, Pandey K, Feng M, Kobayashi S, Sherchand JB. Prevalence of Entamoeba nuttalli infection in wild rhesus macaques in Nepal and characterization of the parasite isolates. Parasitology International. (2013) 62(2):230-5. doi: 10.1016/j.parint.2013.01.004

213. Tachibana H, Yanagi T, Feng M, Bandara KBAT, Kobayashi S, Cheng X, Hirayama K, Rajapakse RPVJ. Isolation and molecular characterization of Entamoeba nuttalli strains showing novel isoenzyme patterns from wild toque macaques in Sri Lanka. Journal of Eukaryotic Microbiology. (2016) 63(2):171-180. doi: 10.1111/jeu.12265

214. Zhang Q, Liu K, Wang C, Luo J, Lu J, He H. Molecular characterization of Entamoeba spp. in wild Taihangshan macaques (Macaca mulatta tcheliensis) in China. Acta Parasitologica. (2019) 64(2):228-231. doi: 10.2478/s11686-019-00026-y

215. Afonso E, Fu R, Dupaix A, Goydadin AC, Yu ZH, Callou C, Villette P, Giraudoux P, Li L. Feeding sites promoting wildlife-related tourism might highly expose the endangered Yunnan snub-nosed monkey (Rhinopithecus bieti) to parasite transmission. Scientific Reports. (2021) 11(1). doi:10.1038/s41598-021-95166-5

216. Tuda J, Feng M, Imada M, Kobayashi S, Cheng X, Tachibana H. Identification of Entamoeba polecki with unique 18S rRNA gene sequences from Celebes crested macaques and pigs in Tangkoko nature reserve, North Sulawesi, Indonesia. The Journal of eukaryotic microbiology. (2016) 63(5):572-7. doi: https://doi.org/10.1111/jeu.12304

217. Lastuti NDR, Suwanti LT, Hastutiek P, Kurniawati DA, Puspitasari H. Molecular detection of Entamoeba Spp in long-tailed macaque (Macaca fascicularis) at Baluran National Park, Indonesia. Malaysian Journal of Medicine and Health Sciences. (2021) 17:85-88.

218. Ekanayake DK, Rajapakse RP, Dubey JP, Dittus WP. Seroprevalence of Toxoplasma gondii in wild toque macaques (Macaca sinica) at Polonnaruwa, Sri Lanka. J Parasitol. (2004) 90(4):870-1. doi: 10.1645/GE-291R

219. Uni S, Kobayashi S, Miyashita M, Kimura N, Kato A, Aimi M, Kimata I, Iseki M, Shoho C. Geographic distribution of Gongylonema pulchrum and Gongylonema macrogubernaculum from Macaca fuscata in Japan. Parasite. (1994) 1(2):127-130. doi: 10.1051/parasite/1994012127

220. Frias L, Stark DJ, Salgado Lynn M, Nathan S, Goossens B, Okamoto M, MacIntosh AJJ. Molecular characterization of nodule worm in a community of Bornean primates. Ecology and Evolution. (2019) 9(7):3937-3945. doi: 10.1002/ece3.5022

221. Horii Y, Imada I, Yanagida T, Usui M, Mori A. Parasite changes and their influence on the body weight of Japanese monkeys (Macaca fuscata fuscata) of the Koshima troop. Primates. (1982) 23(3):416-431. doi: 10.1007/BF02381324

222. Thanchomnang T, Intapan PM, Sanpool O, Rodpai R, Sadaow L, Phosuk I, Somboonpatarakun C, Laymanivong S, Tourtip S, Maleewong W. First molecular identification of Strongyloides fuelleborni in long-tailed macaques in Thailand and Lao People's Democratic Republic reveals considerable genetic diversity. Journal of Helminthology. (2019) 93(5):608-615. doi: 10.1017/S0022149X18000512

223. Hewavithana DK, Wijesinghe MR, Udagama PV. Gastrointestinal parasites of six large mammals in the Wasgomuwa National Park, Sri Lanka. International Journal for Parasitology: Parasites and Wildlife. (2022) 17:1-6. doi: https://doi.org/10.1016/j.ijppaw.2021.11.008

224. Hagiwara K, Tsuge Y, Asakawa M, Kabaya H, Okamoto M, Miyasho T, Taniyama H, Ishihara C, de la Torre JC, Ikuta K. Borna disease virus RNA detected in Japanese macaques (Macaca fuscata). Primates. (2008) 49(1):57-64. doi: 10.1007/s10329-007-0068-8

225. Kosoltanapiwat N, van der Hoek L, Kinsella CM, Tongshoob J, Prasittichai L, Klein M, Jebbink MF, Deijs M, Reamtong O, Boonnak K, Khongsiri W, Phadungsombat J, Tongthainan D, Tulayakul P, Yindee M. A novel simian Adenovirus associating with human Adeno-virus species G isolated from long-tailed macaque feces. Viruses. (2023) 15(6). doi: 10.3390/v15061371

226. Tan B, Wu LJ, Yang XL, Li B, Zhang W, Lei YS, Li Y, Yang GX, Chen J, Chen G, Wang HZ, Shi ZL. Isolation and characterization of adenoviruses infecting endangered golden snub-nosed monkeys (Rhinopithecus roxellana). Virology Journal. (2016) 13(1):190. doi: 10.1186/s12985-016-0648-6

227. Karlsson EA, Engel GA, Feeroz MM, San S, Rompis A, Lee BP, Shaw E, Oh G, Schillaci MA, Grant R, Heidrich J, Schultz-Cherry S, Jones-Engel L. Influenza virus infection in nonhuman primates. Emerg Infect Dis. (2012) 18(10):1672-5. doi:10.3201/eid1810.120214

228. Xin YY, Li LL, Ao YY, Xie ZP, Li JS, Duan ZJ, Yu JM, Zhang B. A novel astrovirus identified in wild rhesus monkey feces in China. Archives of Virology. (2019) 164(9):2385-2388. doi: 10.1007/s00705-019-04319-5

229. Ao YY, Yu JM, Zhang CY, Xin YY, Li LL, Duan ZJ. Identification of a novel enterovirus species in rhesus macaque in China. Scientific Reports. (2016) 6. doi: 10.1038/srep28526.

230. Islam A, Hossain ME, Haider N, Rostal MK, Mukharjee SK, Ferdous J, Miah M, Rahman M, Daszak P, Rahman MZ, Epstein JH. Molecular characterization of group A rotavirus from rhesus macaques (Macaca mulatta) at human–wildlife interfaces in Bangladesh. Transboundary and Emerging Diseases. (2020) 67(2):956-966. doi: 10.1111/tbed.13431

231. Sam IC, Chua CL, Rovie-Ryan JJ, Fu JY, Tong C, Sitam FT, Chan YF. Chikungunya virus in macaques, Malaysia. Emerg Infect Dis. (2015) 21(9):1683-5. doi:10.3201/eid2109.150439

232. Chua CL, Chan YF, Andu E, Rovie-Ryan JJ, Sitam FT, Verasahib K, Sam IC. Little evidence of Zika virus infection in wild long-tailed macaques, Peninsular Malaysia. Emerg Infect Dis. (2019) 25(2):374-376. doi:10.3201/eid2502.180258

233. Ferdous FB, Islam MS, Ullah MA, Rana ML, Punom SA, Neloy FH, Chowdhury MNU, Hassan J, Siddique MP, Saha S, Rahman MT. Antimicrobial resistance profiles, virulence determinants, and biofilm formation in Enterococci isolated from rhesus macaques (Macaca mulatta): A potential threat for wildlife in Bangladesh? Animals. (2023) 13(14):2268. doi:10.3390/ani13142268

234. Singh SV, Singh AV, Singh PK, Kumar A, Singh B. Molecular identification and characterization of Mycobacterium avium subspecies paratuberculosis in free living non-human primate (Rhesus macaques) from North India. Comparative Immunology, Microbiology and Infectious Diseases. (2011) 34(3):267-271. doi: https://doi.org/10.1016/j.cimid.2010.12.004
