# Supplementary material for: Zoonotic pathogens in wild Asian primates: a systematic review highlighting research gaps
Source: Front Vet Sci. 2024 Jun 27;11:1386180. doi: 10.3389/fvets.2024.1386180 (PMC11238137; doi:10.3389/fvets.2024.1386180)
Supplement: Supplementary file 2 [file Table_1.DOC]

Supplementary Material

Zoonotic pathogens of wild Asian primates in different habitat types: A systematic review

Laurie Patouillat^1,2*^, Alain Hambuckers^1^, Sena Adi Subrata^3^, Mutien-Marie Garigliany^2^†, Fany Brotcorne^1^†

*** Correspondence:** Laurie Patouillat ; laurie.patouillat@uliege.be

# Supplementary Figures and Tables

## Supplementary Tables

**Table S1** : Distribution in primate hosts of zoonotic Protozoa in interface habitats and their transmission routes

|  | Urban habitat | Rural habitat | Forest habitat | Main transmission route | References |
| --- | --- | --- | --- | --- | --- |
| *Hepatocystis sp* |  |  | *Macaca fascicularis*  *Macaca nemestrina* | Vector Borne | (57)(64) |
| *Plasmodium sp* | *Macaca fascicularis*  *Macaca nemestrina* | *Macaca fascicularis* | *Macaca fascicularis*  *Macaca nemestrina*  *Macaca hecki* |  | (63) (64) (122) (207)(208) |
| *Plasmodium cynomolgi* | *Macaca fascicularis*  *Macaca nemestrina* | *Macaca fascicularis*  *Macaca nemestrina* | *Macaca fascicularis*  *Macaca nemestrina*  *Macaca sinica*  *Macaca radiata*  *Macaca leonina*  *Macaca arctoides*  *Presbytis entellus* |  | (52)(53)(54)(55)  (56)(58)(59)(60)  (62)(63)(65) |
| *Plasmodium falciparum* | *Macaca mulatta*  *Macaca radiata* |  | *Macaca mulatta*  *Macaca radiata* |  | (55) |
| *Plasmodium fieldi* | *Macaca fascicularis*  *Macaca nemestrina* | *Macaca fascicularis*  *Macaca nemestrina* | *Macaca fascicularis*  *Macaca nemestrina*  *Macaca leonina*  *Macaca arctoides* |  | (52)(53)(56)(58)  (60)(65) |
| *Plasmodium inui* | *Macaca fascicularis*  *Macaca nemestrina* | *Macaca fascicularis*  *Macaca nemestrina* | *Macaca cyclopis*  *Macaca fascicularis*  *Macaca nemestrina*  *Macaca leonina*  *Macaca arctoides*  *Macaca radiata* |  | (52)(53)(55)(56)  (57)(58)(59)(60)  (61)(62) (64)(65) |
| *Plasmodium knowlesi* | *Macaca fascicularis*  *Macaca nemestrina* | *Macaca fascicularis*  *Macaca nemestrina* | *Macaca fascicularis*  *Macaca nemestrina*  *Macaca arctoides* |  | (52)(53)(56)(58)  (59)(60)(63)(64)  (65)(66)(67)(68) |
| *Balantidium sp* | *Macaca fascicularis*  *Macaca sinica*  *Macaca mulatta* | *Macaca fascicularis*  *Macaca sinica*  *Macaca mulatta*  *Trachypithecus johnii* | *Macaca fascicularis*  *Macaca sinica*  *Pongo pygmaeus*  *Trachypithecus johnii* | Fecal/Oral | (23)(24)(73)(87)  (105)(107) |
| *Balantidium coli* | *Macaca mulatta*  *Macaca sinica* | *Macaca mulatta*  *Macaca sinica*  *Macaca silenus*  *Macaca fascicularis*  *Macaca thibetana*  *Macaca maura* | *Macaca fascicularis*  *Macaca mulatta*  *Macaca sinica*  *Macaca silenus*  *Macaca nigra*  *Macaca maura*  *Pongo pygmaeus*  *Pongo abelii*  *Hylobates lar* |  | (71)(78)(79)(80)  (84)(85)(86)(93)  (94)(95)(98)(109)(182)(209) |
| *Blastocystis sp* | *Macaca fascicularis*  *Macaca nemestrina*  *Macaca*  *mulatta*  *Trachypithecus*  *cristatus*  *Trachypithecus*  *obscurus*  *Nasalis larvatus* | *Macaca fascicularis*  *Macaca nemestrina*  *Macaca mulatta*  *Macaca thibetana*  *Macaca maura*  *Trachypithecus*  *cristatus*  *Trachypithecus*  *obscurus*  *Nasalis larvatus* | *Macaca fascicularis*  *Macaca nemestrina*  *Macaca mulatta*  *Macaca maura*  *Trachypithecus*  *cristatus*  *Trachypithecus*  *obscurus*  *Nasalis larvatus*  *Pongo pygmaeus* |  | (24)(76)(109)  (105)(182)(183)  (184)(185)(210) |
| *Blastocystis hominis* | *Macaca mulatta* |  | *Macaca mulatta* |  | (209) |
| *Buxtonella sp* | *Macaca sinica* |  |  |  | (73) |
| *Chilomastix mesnili* | *Macaca mulatta* | *Macaca maura* | *Macaca mulatta*  *Macaca maura*  *Pongo pygmaeus* |  | (71)(109)(209) |
| *Chilomastix sp* | *Macaca sinica* | *Macaca sinica* | *Macaca sinica* |  | (80) |
| *Coccidia sp* | *Macaca radiata* | *Macaca silenus*  *Macaca fascicularis*  *Trachypithecus johnii* | *Macaca silenus*  *Macaca radiata*  *Macaca fascicularis*  *Trachypithecus johnii* |  | (77)(84)(94)(107) |
| *Cryptosporidia sp* | *Macaca fascicularis* | *Macaca fascicularis* | *Macaca fascicularis* |  | (24) |
| *Cryptosporidium sp* | *Macaca fascicularis*  *Macaca nemestrina*  *Macaca*  *mulatta*  *Macaca sinica*  *Semnopithecus*  *entellus*  *Semnopithecus vetulus*  *Trachypithecus*  *cristatus*  *Trachypithecus*  *obscurus*  *Nasalis larvatus* | *Macaca fascicularis*  *Macaca nemestrina*  *Macaca*  *mulatta*  *Macaca sinica*  *Semnopithecus*  *entellus*  *Semnopithecus vetulus*  *Trachypithecus*  *cristatus*  *Trachypithecus*  *obscurus*  *Nasalis larvatus* | *Macaca fascicularis*  *Macaca nemestrina*  *Macaca*  *mulatta*  *Macaca sinica*  *Semnopithecus*  *entellus*  *Semnopithecus vetulus*  *Trachypithecus*  *cristatus*  *Trachypithecus*  *obscurus*  *Nasalis larvatus*  *Hylobates lar* |  | (24)(73)(76)(80)  (85)(86)(98)(103)(113)(186)(187) |
| *Cryptosporidium hominis* |  | *Macaca mulatta* |  |  | (188) |
| *Cryptosporidium parvum* |  | *Macaca mulatta* | *Macaca mulatta* |  | (132)(188) |
| *Cyclospora sp* | *Macaca fascicularis*  *Macaca nemestrina*  *Macaca*  *mulatta*  *Trachypithecus*  *cristatus*  *Trachypithecus*  *obscurus*  *Nasalis larvatus* | *Macaca fascicularis*  *Macaca nemestrina*  *Trachypithecus*  *cristatus*  *Trachypithecus*  *Obscurus*  *Trachypithecus johnii*  *Nasalis larvatus* | *Macaca fascicularis*  *Macaca nemestrina*  *Macaca*  *mulatta*  *Trachypithecus*  *cristatus*  *Trachypithecus*  *Obscurus*  *Trachypithecus johnii*  *Nasalis larvatus* |  | (76)(107)(113)  (209) |
| *Dientamoeba fragilis* | *Macaca fascicularis* |  |  |  | (184) |
| *Eimeria sp* | *Macaca sinica* |  |  |  | (73) |
| *Endolimax sp* | *Macaca fascicularis*  *Macaca sinica* | *Macaca fascicularis* | *Macaca fascicularis*  *Macaca sinica* |  | (23)(24) |
| *Endolimax nana* | *Macaca fascicularis*  *Macaca mulatta* |  | *Pongo pygmaeus*  *Macaca mulatta* |  | (184)(105)(209) |
| *Entamoeba chattoni* | *Macaca cyclopis*  *Macaca fascicularis*  *Macaca mulatta*  *Macaca sinica* | *Macaca mulatta* | *Macaca cyclopis*  *Macaca fascicularis*  *Macaca mulatta*  *Macaca sinica* |  | (189)(190)(209)  (211)(212)(213)  (214) |
| *Entamoeba coli* | *Macaca mulatta*  *Macaca sinica*  *Macaca radiata*  *Macaca cyclopis*  *Macaca fascicularis*  *Semnopithecus entellus*  *Semnopithecus vetulus* | *Macaca mulatta*  *Macaca sinica*  *Macaca cyclopis*  *Macaca fascicularis*  *Semnopithecus entellus*  *Semnopithecus vetulus* | *Macaca mulatta*  *Macaca sinica*  *Macaca radiata*  *Macaca silenus*  *Macaca fascicularis*  *Macaca cyclopis*  *Pongo pygmaeus*  *Semnopithecus entellus*  *Semnopithecus vetulus* |  | (71)(77)(80)(84)  (86)(94)(96)(98)  (103)(105)(110)  (184)(186)(189)  (190)(209)(211)  (212)(213)(214) |
| *Entamoeba dispar* | *Macaca sinica*  *Macaca fascicularis*  *Macaca mulatta*  *Semnopithecus entellus*  *Semnopithecus vetulus* | *Macaca sinica*  *Macaca mulatta*  *Semnopithecus entellus*  *Semnopithecus vetulus* | *Macaca mulatta*  *Macaca sinica*  *Macaca fuscata*  *Semnopithecus entellus*  *Semnopithecus vetulus*  *Pongo pygmaeus* |  | (80)(133)(184)  (105)(110)(209)  (212)(213)(214) |
| *Entamoeba hartmanni* | *Macaca mulatta*  *Macaca sinica*  *Macaca cyclopis* | *Macaca sinica* | *Macaca mulatta*  *Macaca sinica*  *Macaca cyclopis*  *Macaca fascicularis*  *Pongo pygmaeus* |  | (80)(189)(190)  (105)(209)(214) |
| *Entamoeba hystolitica* | *Macaca sinica*  *Macaca fascicularis*  *Macaca mulatta*  *Semnopithecus entellus*  *Semnopithecus vetulus* | *Macaca sinica*  *Macaca silenus*  *Semnopithecus entellus*  *Semnopithecus vetulus* | *Macaca sinica*  *Macaca silenus*  *Pongo abelii*  *Pongo pygmaeus*  *Semnopithecus entellus*  *Semnopithecus vetulus* |  | (78)(80)(84)(86)  (103)(105)(110)  (184)(191) |
| *Entamoeba moshkovskii* |  |  | *Macaca fascicularis* |  | (190) |
| *Entamoeba nutalli* | *Macaca mulatta*  *Macaca cyclopis*  *Macaca sinica*  *Macaca fascicularis* | *Macaca mulatta* | *Macaca mulatta*  *Macaca cyclopis*  *Macaca sinica*  *Macaca fascicularis* |  | (189)(209)(211)  (212)(213) |
| *Entamoeba pölecki* | *Macaca fascicularis* |  | *Macaca nigra*  *Rhinopithecus bieti* |  | (184)(215)(216) |
| *Entamoeba sp* | *Macaca fascicularis*  *Macaca nemestrina*  *Macaca*  *mulatta*  *Macaca sinica*  *Trachypithecus*  *cristatus*  *Trachypithecus*  *obscurus*  *Nasalis larvatus* | *Macaca fascicularis*  *Macaca nemestrina*  *Macaca*  *mulatta*  *Macaca sinica*  *Macaca thibetana*  *Macaca maura*  *Trachypithecus*  *cristatus*  *Trachypithecus*  *obscurus*  *Nasalis larvatus* | *Macaca fascicularis*  *Macaca nemestrina*  *Macaca*  *mulatta*  *Macaca maura*  *Macaca nigra*  *Trachypithecus*  *cristatus*  *Trachypithecus*  *obscurus*  *Nasalis larvatus*  *Pongo pygmaeus*  *Pongo abelii*  *Presbytis femoralis*  *Rhinopithecus bieti* |  | (71)(77)(80)(84)  (89)(91)(93)(96)  (98)(105)(109)  (182)(184)(186)  (189)(215)(217) |
| *Giardia duodenalis* | *Macaca fascicularis* | *Macaca maura* | *Macaca mulatta*  *Macaca maura*  *Rhinopithecus bieti* |  | (109)(132)(187) |
| *Giardia sp* | *Macaca fascicularis*  *Macaca radiata*  *Macaca mulatta* | *Macaca fascicularis*  *Macaca mulatta* | *Macaca fascicularis*  *Macaca mulatta*  *Macaca radiata*  *Macaca nigra*  *Pongo pygmaeus* |  | (24)(77)(86)(93)  (105)(186)(209) |
| *Iadomoeba buetschlii* | *Macaca fascicularis*  *Macaca mulatta* | *Macaca maura* | *Macaca mulatta Macaca fuscata*  *Macaca maura*  *Pongo pygmaeus* |  | (86)(105)(109)  (133)(184)(209) |
| *Iadomoeba sp* | *Macaca fascicularis*  *Macaca sinica* | *Macaca fascicularis*  *Macaca sinica* | *Macaca fascicularis*  *Macaca sinica* |  | (24)(80) |
| *Isospora sp* | *Macaca fascicularis*  *Macaca sinica*  *Macaca mulatta* | *Macaca fascicularis*  *Macaca sinica* | *Macaca fascicularis*  *Macaca mulatta*  *Macaca nigra* |  | (23)(24)(79)(93) |
| *Parabertiella sp* |  |  | *Presbytis cristatus* |  | (111) |
| *Retortamonas sp* | *Macaca fascicularis* | *Macaca fascicularis* | *Macaca fascicularis* |  | (24) |
| *Trichomonas sp* |  |  |  |  |  |
| *Toxoplasma sp* | *Macaca mulatta* |  |  |  | (89) |
| *Toxoplasma gondii* | *Macaca sinica* | *Macaca sinica* | *Macaca sinica* |  | (218) |

**Supplementary Table S1 :** List of zoonotic protozoa found in each habitat type defined by color (blue = urban, orange = rural, green = forest). Each colored box represents the presence of a pathogen in an interface.

**Table S2** : Distribution in primate hosts of zoonotic Nematode gastrointestinal parasites according to the interface habitats

|  | Urban habitat | Rural habitat | Forest habitat | Main transmission route | References |
| --- | --- | --- | --- | --- | --- |
| *Abbreviata caucasica* |  |  | *Pongo abelii* | Fecal/Oral | (78) |
| *Anatrichosoma sp* |  | *Nasalis larvatus*  *Macaca fascicularis*  *Macaca nemestrina* | *Nasalis larvatus*  *Macaca fascicularis*  *Macaca nemestrina*  *Macaca hecki* |  | (73)(75)(92)(104)  (208) |
| *Ancylostoma duodenale* |  | *Macaca thibetana* | *Macaca thibetana* |  | (82)(182) |
| *Ancylostoma sp* | *Macaca mulatta*  *Macaca fascicularis*  *Macaca sinica* | *Macaca silenus*  *Macaca fascicularis*  *Macaca mulatta* | *Macaca silenus*  *Macaca fascicularis*  *Macaca mulatta*  *Macaca nigra*  *Pongo abelii* |  | (24)(73)(78)(79)  (89)(91)(93)(100)  (103) |
| *Ascarid sp* | *Macaca mulatta*  *Macaca fascicularis* | *Macaca mulatta* | *Macaca mulatta*  *Macaca fascicularis*  *Macaca nigra* |  | (74)(86)(93)(98) |
| *Ascaris lumbricoides* |  | *Macaca thibetana* | *Macaca thibetana*  *Semnopithecus vetullus*  *Nasalis larvatus* |  | (81)(82)(104)  (182) |
| *Ascaris sp* | *Macaca fascicularis*  *Macaca nemestrina*  *Macaca*  *mulatta*  *Macaca radiata*  *Macaca sinica*  *Trachypithecus*  *cristatus*  *Trachypithecus*  *obscurus*  *Nasalis larvatus* | *Macaca fascicularis*  *Macaca nemestrina*  *Macaca silenus*  *Macaca sinica*  *Macaca mulatta*  *Trachypithecus*  *cristatus*  *Trachypithecus*  *obscurus*  *Trachypithecus johnii*  *Nasalis larvatus* | *Macaca fascicularis*  *Macaca nemestrina*  *Macaca mulatta*  *Macaca silenus*  *Macaca radiata*  *Trachypithecus*  *cristatus*  *Trachypithecus*  *obscurus*  *Trachypithecus johnii*  *Nasalis larvatus*  *Pongo abelii*  *Hylobates lar* |  | (23)(24)(72)(73)  (76)(77)(78)(84)  (85)(80)(89)(91)  (101)(106)(107)  (113) |
| *Bertiella sp* |  | *Macaca sinica* | *Presbytis cristatus* |  | (23)(111) |
| *Bunostomum sp* |  | *Macaca silenus*  *Macaca fascicularis* | *Macaca silenus*  *Trachypithecus johnii* |  | (84)(94)(100)  (107) |
| *Capillaria hepatica* |  |  | *Macaca thibetana* |  | (82) |
| *Capillaria sp* | *Macaca fascicularis*  *Macaca mulatta*  *Macaca sinica*  *Trachypithecus cristatus*  *Trachypithecus obscurus*  *Nasalis larvatus* | *Macaca fascicularis*  *Trachypithecus cristatus*  *Trachypithecus obscurus*  *Nasalis larvatus* | *Macaca fascicularis*  *Macaca mulatta*  *Trachypithecus cristatus*  *Trachypithecus obscurus*  *Nasalis larvatus Nycticebus menagensis*  *Cephalopachus bancanus*  *Tarsius syrichta* |  | (73)(75)(76)(79)  (89)(114) |
| *Enterobius sp* | *Macaca fascicularis*  *Macaca nemestrina*  *Macaca mulatta*  *Macaca sinica*  *Trachypithecus cristatus*  *Trachypithecus obscurus*  *Nasalis larvatus* | *Macaca fascicularis*  *Macaca nemestrina*  *Macaca sinica*  *Trachypithecus cristatus*  *Trachypithecus obscurus*  *Nasalis larvatus* | *Macaca fascicularis*  *Macaca nemestrina*  *Macaca mulatta*  *Macaca sinica*  *Macaca fuscata*  *Trachypithecus cristatus*  *Trachypithecus obscurus*  *Trachypithecus johnii*  *Nasalis larvatus*  *Pongo pygmaeus*  *Pongo abelii*  *Presbytis entellus*  *Presbytis senex*  *Presbytis cristatus* |  | (23)(24)(73)(76)  (78)(79)(80)(89)  (92)(95)(104)  (105)(107)(108)  (111)(113)(133) |
| *Enterobius vermicularis* |  | *Macaca silenus*  *Macaca fascicularis*  *Macaca thibetana* | *Macaca silenus* |  | (84)(94)(182) |
| *Gongylonema pulchrum* |  |  | *Macaca thibetana*  *Macaca fuscata* |  | (82)(192)(219) |
| *Gongylonema sp* |  | *Macaca thibetana*  *Trachypithecus johnii* | *Macaca thibetana*  *Trachypithecus johnii* |  | (107)(182) |
| *Haemonchus sp* |  | *Macaca silenus*  *Macaca fascicularis* | *Macaca silenus*  *Macaca nigra* |  | (77)(84)(93)(94) |
| *Heterakis sp* | *Macaca fascicularis*  *Macaca nemestrina*  *Trachypithecus cristatus*  *Trachypithecus obscurus*  *Nasalis larvatus* | *Macaca fascicularis*  *Macaca nemestrina*  *Trachypithecus cristatus*  *Trachypithecus obscurus*  *Nasalis larvatus* | *Macaca fascicularis*  *Macaca nemestrina*  *Trachypithecus cristatus*  *Trachypithecus obscurus*  *Nasalis larvatus* |  | (76) |
| *Hookworm* | *Macaca fascicularis*  *Macaca nemestrina*  *Macaca mulatta*  *Macaca sinica*  *Trachypithecus cristatus*  *Trachypithecus obscurus*  *Nasalis larvatus*  *Semnopithecus entellus* | *Macaca fascicularis*  *Macaca nemestrina*  *Macaca mulatta*  *Macaca sinica*  *Trachypithecus cristatus*  *Trachypithecus obscurus*  *Nasalis larvatus*  *Semnopithecus entellus* | *Macaca fascicularis*  *Macaca nemestrina*  *Macaca mulatta*  *Macaca fuscata*  *Macaca sinica*  *Trachypithecus cristatus*  *Trachypithecus obscurus*  *Nasalis larvatus*  *Semnopithecus entellus*  *Pongo pygmaeus* |  | (23)(72)(74)(76)  (83)(86)(98)(99)  (101)(105)(106)  (110)(133) |
| *Metastrongylus sp* |  | *Macaca silenus* | *Macaca silenus* |  | (84) |
| *Necator sp* |  |  | *Hylobates lar* |  | (85) |
| *Oesophagostomum aculeatum* |  | *Macaca fuscata* | *Macaca fascicularis Macaca fuscata*  *Macaca sinica*  *Presbytis entellus*  *Trachypithecus cristatus*  *Nasalis larvatus*  *Pongo pygmaeus* |  | (108)(192)(193)  (220)(221) |
| *Oesophagostomum apiostomum* |  |  | *Macaca thibetana* |  | (82) |
| *Oesophagostomum sp* | *Macaca fascicularis*  *Macaca nemestrina*  *Macaca*  *mulatta*  *Macaca sinica*  *Trachypithecus*  *cristatus*  *Trachypithecus*  *obscurus*  *Nasalis larvatus*  *Semnopithecus vetullus* | *Macaca fascicularis*  *Macaca nemestrina*  *Macaca*  *silenus*  *Macaca sinica*  *Trachypithecus*  *cristatus*  *Trachypithecus*  *obscurus*  *Trachypithecus johnii*  *Nasalis larvatus*  *Semnopithecus vetullus* | *Macaca fascicularis*  *Macaca nemestrina*  *Macaca*  *silenus*  *Macaca mulatta*  *Macaca hecki*  *Trachypithecus*  *cristatus*  *Trachypithecus*  *obscurus*  *Trachypithecus*  *auratus*  *Trachypithecus johnii*  *Nasalis larvatus*  *Semnopithecus vetullus*  *Presbytis femoralis*  *Presbytis cristatus*  *Pongo abelii*  *Hylobates moloch* |  | (23)(73)(76)(78)  (79)(81)(84)(88)  (94)(96)(97)(101)  (102)(104)(107)  (111)(208) |
| *Oxyurida sp* |  | *Nasalis larvatus* | *Macaca hecki*  *Nycticebus menagensis*  *Cephalopachus bancanus*  *Nasalis larvatus*  *Tarsius syrichta* |  | (75)(101)(114)  (208) |
| *Oxyuris sp* | *Macaca fascicularis*  *Macaca nemestrina*  *Trachypithecus*  *cristatus*  *Trachypithecus*  *obscurus*  *Nasalis larvatus* | *Macaca fascicularis*  *Macaca nemestrina*  *Trachypithecus*  *cristatus*  *Trachypithecus*  *Obscurus*  *Nasalis larvatus* | *Macaca fascicularis*  *Macaca nemestrina*  *Trachypithecus*  *cristatus*  *Trachypithecus*  *Obscurus*  *Nasalis larvatus* |  | (76) |
| *Parascaris sp* | *Macaca fascicularis*  *Macaca nemestrina*  *Trachypithecus*  *cristatus*  *Trachypithecus*  *obscurus*  *Nasalis larvatus* | *Macaca fascicularis*  *Macaca nemestrina*  *Trachypithecus*  *cristatus*  *Trachypithecus*  *obscurus*  *Nasalis larvatus* | *Macaca fascicularis*  *Macaca nemestrina*  *Trachypithecus*  *cristatus*  *Trachypithecus*  *obscurus*  *Nasalis larvatus* |  | (76) |
| *Physaloptera sp* | *Macaca fascicularis*  *Macaca nemestrina*  *Macaca mulatta*  *Macaca sinica*  *Trachypithecus*  *cristatus*  *Trachypithecus*  *obscurus*  *Nasalis larvatus* | *Macaca fascicularis*  *Macaca nemestrina*  *Macaca thibetana*  *Trachypithecus*  *cristatus*  *Trachypithecus*  *obscurus*  *Nasalis larvatus* | *Macaca fascicularis*  *Macaca nemestrina*  *Macaca mulatta*  *Macaca sinica*  *Macaca hecki*  *Trachypithecus*  *cristatus*  *Trachypithecus*  *obscurus*  *Nasalis larvatus* |  | (73)(76)(79)(86)  (101)(108)(182)  (208) |
| *Rhabditis sp* | *Macaca fascicularis*  *Macaca nemestrina*  *Macaca thibetana*  *Trachypithecus*  *cristatus*  *Trachypithecus*  *obscurus*  *Nasalis larvatus* | *Macaca fascicularis*  *Macaca nemestrina*  *Trachypithecus*  *cristatus*  *Trachypithecus*  *obscurus*  *Nasalis larvatus* | *Macaca fascicularis*  *Macaca nemestrina*  *Trachypithecus*  *cristatus*  *Trachypithecus*  *obscurus*  *Nasalis larvatus* |  | (76)(82) |
| *Spirurida sp* |  | *Macaca fascicularis*  *Macaca nemestrina*  *Trachypithecus*  *cristatus*  *Nasalis larvatus* | *Macaca fascicularis*  *Macaca nemestrina*  *Trachypithecus*  *cristatus*  *Nasalis larvatus*  *Nycticebus menagensis*  *Pongo pygmaeus* |  | (75) |
| *Spiruroid type eggs* | *Macaca sinica* | *Macaca silenus*  *Macaca sinica* | *Macaca silenus*  *Macaca sinica* |  | (80)(84) |
| *Strongyle type eggs* | *Macaca sinica*  *Macaca mulatta* | *Macaca sinica*  *Macaca mulatta* | *Macaca sinica*  *Hylobates lar*  *Tarsius syrichta* |  | (80)(85)(86)(91)  (114) |
| *Strongylida sp* |  | *Macaca maura* | *Pongo pygmaeus*  *Pongo abelii*  *Nycticebus menagensis*  *Cephalopachus bancanus*  *Macaca fascicularis*  *Macaca maura*  *Macaca nemestrina*  *Trachypithecus*  *cristatus*  *Nasalis larvatus* |  | (75)(78)(104)  (109) |
| *Strongyloides fuelleborni* | *Macaca fascicularis*  *Macaca mulatta* | *Macaca fuscata* | *Macaca fascicularis*  *Macaca fuscata*  *Macaca mulatta*  *Macaca sinica*  *Macaca hecki*  *Pongo pygmaeus*  *Presbytis entellus*  *Hylobates lar* |  | (71)(85)(108)  (192)(193)(194)  (208)(209)(221)  (222) |
| *Strongyloides sp* | *Macaca fascicularis*  *Macaca nemestrina*  *Macaca mulatta*  *Macaca sinica*  *Macaca radiata*  *Trachypithecus*  *cristatus*  *Trachypithecus*  *obscurus*  *Nasalis larvatus*  *Semnopithecus vetullus* | *Macaca fascicularis*  *Macaca nemestrina*  *Macaca sinica*  *Macaca silenus*  *Macaca mulatta*  *Trachypithecus*  *cristatus*  *Trachypithecus*  *obscurus*  *Trachypithecus johnii*  *Nasalis larvatus*  *Semnopithecus vetullus* | *Macaca fascicularis*  *Macaca nemestrina*  *Macaca mulatta*  *Macaca sinica*  *Macaca silenus*  *Macaca radiata*  *Macaca nigra*  *Hylobates moloch*  *Presbytis femoralis*  *Pongo pygmaeus*  *Pongo abelii*  *Trachypithecus*  *cristatus*  *Trachypithecus*  *Obscurus*  *Trachypithecus auratus*  *Trachypithecus johnii*  *Nasalis larvatus*  *Semnopithecus vetullus*  *Nycticebus menagensis*  *Cephalopachus bancanus* |  | (23)(71)(72)(73)  (74)(75)(76)(77)  (78)(79)(80)(81)  (86)(87)(88)(89)  (90)(91)(92)(93)  (95)(96)(97)(98)  (99)(100)(101)  (102)(103)(104)  (105)(106)(107) |
| *Strongyloides stercoralis* | *Macaca fascicularis*  *Macaca nemestrina*  *Trachypithecus*  *cristatus*  *Trachypithecus*  *obscurus*  *Nasalis larvatus* | *Macaca fascicularis*  *Macaca nemestrina*  *Macaca silenus*  *Trachypithecus*  *cristatus*  *Trachypithecus*  *obscurus*  *Nasalis larvatus* | *Macaca fascicularis*  *Macaca nemestrina*  *Macaca silenus*  *Macaca thibetana*  *Trachypithecus*  *cristatus*  *Trachypithecus*  *obscurus*  *Nasalis larvatus* |  | (76)(82)(83)(84)  (94)(101) |
| *Ternidens sp* |  | *Nasalis larvatus* | *Nasalis larvatus*  *Pongo abelii*  *Hylobates lar* |  | (78)(85)(92)(104) |
| *Ternidens deminutus* |  |  | *Pongo pygmaeus* |  | (220) |
| *Toxocara sp* | *Macaca mulatta* | *Macaca silenus*  *Macaca mulatta*  *Macaca fascicularis* |  |  | (84)(86)(87)(94) |
| *Trichostrongylus sp* | *Macaca fascicularis*  *Macaca nemestrina*  *Macaca mulatta*  *Macaca sinica*  *Trachypithecus*  *cristatus*  *Trachypithecus*  *obscurus*  *Nasalis larvatus*  *Semnopithecus vetulus* | *Macaca fascicularis*  *Macaca nemestrina*  *Macaca silenus*  *Trachypithecus*  *cristatus*  *Trachypithecus*  *obscurus*  *Trachypithecus johnii*  *Nasalis larvatus*  *Semnopithecus vetulus* | *Macaca fascicularis*  *Macaca nemestrina*  *Macaca mulatta*  *Macaca silenus*  *Macaca thibetana*  *Macaca nigra*  *Macaca sinica*  *Pongo abelii*  *Pongo pygmaeus*  *Trachypithecus*  *cristatus*  *Trachypithecus johnii*  *Trachypithecus*  *obscurus*  *Trachypithecus auratus*  *Nasalis larvatus*  *Semnopithecus vetulus*  *Hylobates lar*  *Hylobates moloch* |  | (23)(24)(76)(78)  (79)(81)(82)(83)  (84)(85)(86)(87)  (88)(90)(92)(93)  (94)(101)(102)  (104)(105)(107)  (108) |
| *Trichurid type egg* | *Macaca mulatta* |  |  |  | (103) |
| *Trichuris sp* | *Macaca fascicularis*  *Macaca nemestrina*  *Macaca mulatta*  *Macaca sinica*  *Trachypithecus*  *cristatus*  *Trachypithecus*  *obscurus*  *Nasalis larvatus*  *Semnopithecus vetulus*  *Semnopithecus entellus* | *Macaca fascicularis*  *Macaca maura*  *Macaca nemestrina*  *Macaca mulatta*  *Macaca sinica*  *Macaca silenus*  *Trachypithecus*  *cristatus*  *Trachypithecus*  *obscurus*  *Nasalis larvatus*  *Semnopithecus vetulus*  *Semnopithecus entellus* | *Macaca fascicularis*  *Macaca maura*  *Macaca nemestrina*  *Macaca mulatta*  *Macaca sinica*  *Macaca silenus*  *Macaca nigra*  *Trachypithecus*  *cristatus*  *Trachypithecus*  *obscurus*  *Trachypithecus auratus*  *Nasalis larvatus*  *Pongo pygmaeus*  *Pongo abelii*  *Presbytis cristatus*  *Hylobates lar*  *Hylobates moloch*  *Nycticebus menagensis*  *Semnopithecus vetulus*  *Semnopithecus entellus* |  | (23)(24)(72)(73)  (74)(75)(76)(78)  (79)(80)(84)(85)  (86)(87)(88)(89)  (90)(91)(92)(93)  (94)(95)(98)(99)  (100)(102)(104)  (105)(106)(109)  (110)(111) |
| *Trichuris trichuria* | *Macaca fascicularis*  *Macaca mulatta*  *Macaca nemestrina*  *Trachypithecus*  *cristatus*  *Trachypithecus*  *obscurus*  *Nasalis larvatus*  *Semnopithecus vetullus* | *Macaca fascicularis*  *Macaca nemestrina*  *Macaca fuscata*  *Trachypithecus*  *cristatus*  *Trachypithecus*  *Obscurus*  *Trachypithecus johnii*  *Nasalis larvatus*  *Semnopithecus vetullus* | *Macaca fascicularis*  *Macaca mulatta*  *Macaca nemestrina*  *Macaca fuscata*  *Macaca thibetana*  *Macaca sinica*  *Macaca hecki*  *Hylobates lar*  *Presbytis entellus*  *Presbytis senex*  *Trachypithecus*  *cristatus*  *Trachypithecus*  *Obscurus*  *Trachypithecus johnii*  *Nasalis larvatus*  *Semnopithecus vetullus* |  | (76)(81)(82)(96)  (101)(107)(108)  (133)(187)(192)  (193)(208)(209)  (221) |

**Supplementary Table S2 :** List of zoonotic gastrointestinal nematodes found in each habitat type defined by color (blue = urban, orange = rural, green = forest). Each colored box represents the presence of a pathogen in an interface.

**Table S3**: Distribution in primate hosts of zoonotic Cestode gastrointestinal parasites by interface habitats

|  | Urban habitat | Rural habitat | Forest habitat | Main transmission route | References |
| --- | --- | --- | --- | --- | --- |
| *Diphyllobthrium sp* | *Macaca fascicularis*  *Macaca nemestrina*  *Macaca radiata*  *Trachypithecus*  *cristatus*  *Trachypithecus*  *obscurus*  *Nasalis larvatus* | *Macaca fascicularis*  *Macaca nemestrina*  *Macaca silenus*  *Macaca sinica*  *Trachypithecus*  *cristatus*  *Trachypithecus*  *obscurus*  *Nasalis larvatus* | *Macaca fascicularis*  *Macaca nemestrina*  *Macaca silenus*  *Macaca radiata*  *Macaca nigra*  *Trachypithecus*  *cristatus*  *Trachypithecus*  *obscurus*  *Nasalis larvatus* | Fecal/Oral | (23)(76)(77)(84)  (93)(101) |
| *Dipylidium caninum* | *Macaca radiata* | *Macaca silenus* | *Macaca radiata*  *Macaca silenus* |  | (77)(84) |
| *Echinococcus sp* |  |  | *Macaca nigra* |  | (93) |
| *Hymenolepsis diminuta* | *Macaca fascicularis* |  |  |  | (187) |
| *Hymenolepsis nana* | *Macaca mulatta* |  | *Trachypithecus johnii* |  | (87)(107) |
| *Hymenolepsis sp* | *Macaca fascicularis*  *Macaca nemestrina*  *Trachypithecus*  *cristatus*  *Trachypithecus*  *obscurus*  *Nasalis larvatus* | *Macaca fascicularis*  *Macaca nemestrina*  *Macaca mulatta*  *Trachypithecus*  *cristatus*  *Trachypithecus*  *obscurus*  *Nasalis larvatus* | *Macaca fascicularis*  *Macaca nemestrina*  *Macaca sinica*  *Macaca nigra*  *Trachypithecus*  *cristatus*  *Trachypithecus*  *obscurus*  *Nasalis larvatus*  *Presbytis entellus*  *Pongo pygmaeus*  *Tarsius syrichta* |  | (23)(76)(91)(93)  (101)(105)(108)  (114) |
| *Moniezia sp* | *Macaca mulatta* |  |  |  | (87) |
| *Taenia sp* | *Macaca fascicularis*  *Macaca mulatta* | *Macaca fascicularis* | *Macaca fascicularis*  *Macaca mulatta* |  | (24)(83)(87)(89)  (113) |

**Supplementary Table S3 :** List of zoonotic gastrointestinal cestodes found in each habitat type defined by color (blue = urban, orange = rural, green = forest). Each colored box represents the presence of a pathogen in an interface.

**Table S4** : Distribution in primate hosts of zoonotic Trematode gastrointestinal parasites by interface habitats

|  | Urban habitat | Rural habitat | Forest habitat | Main transmission route | References |
| --- | --- | --- | --- | --- | --- |
| *Alaria sp* | *Macaca fascicularis* |  | *Macaca fascicularis* | Fecal/Oral | (24) |
| *Clonorchis sp* | *Macaca fascicularis*  *Macaca nemestrina* | *Macaca fascicularis*  *Macaca nemestrina* | *Macaca fascicularis*  *Macaca nemestrina* |  | (76) |
| *Dicrocoeliidae sp* |  |  | *Pongo abelii*  *Hylobates lar* |  | (78)(85) |
| *Digenean type egg* |  |  | *Semnopithecus priam* |  | (223) |
| *Fasciola sp* | *Macaca fascicularis*  *Macaca nemestrina*  *Trachypithecus*  *cristatus*  *Trachypithecus*  *obscurus*  *Nasalis larvatus* | *Macaca fascicularis*  *Macaca nemestrina*  *Trachypithecus*  *cristatus*  *Trachypithecus*  *obscurus*  *Nasalis larvatus* | *Macaca fascicularis*  *Macaca nemestrina*  *Trachypithecus*  *cristatus*  *Trachypithecus*  *obscurus*  *Nasalis larvatus* |  | (76)(101) |
| *Gasterodiscoides sp* |  |  | *Pongo abelii*  *Presbytis cristatus* |  | (78)(111) |
| *Minute intestinal flukes* | *Macaca fascicularis* | *Macaca fascicularis* | *Macaca fascicularis* |  | (83) |
| *Opisthorchis viverrini* |  |  |  |  |  |
| *Paragonimus sp* | *Macaca fascicularis*  *Macaca mulatta* | *Macaca fascicularis* | *Macaca fascicularis* |  | (24)(89) |
| *Schistosoma sp* | *Macaca fascicularis*  *Macaca nemestrina*  *Macaca mulatta* | *Macaca fascicularis*  *Macaca nemestrina*  *Trachypithecus johnii* | *Macaca fascicularis*  *Macaca nemestrina*  *Nasalis larvatus* |  | (76)(89)(101)  (107) |
| *Schistosoma japanicum* |  |  | *Macaca nigra* |  | (93) |
| *Schistosoma mekongi* |  |  |  |  |  |
| *Watsonius sp* | *Macaca mulatta* |  | *Macaca hecki* |  | (89)(208) |

**Supplementary Table S4 :** List of zoonotic gastrointestinal trematodes found in each habitat type defined by color (blue = urban, orange = rural, green = forest). Each colored box represents the presence of a pathogen in an interface.

**Table S5** : Distribution in primate hosts of zoonotic Viruses in interface habitats and their transmission routes

|  | Urban habitat | Rural habitat | Forest habitat | Main transmission route | References |
| --- | --- | --- | --- | --- | --- |
| Adult T-cell Leukemia |  |  | *Macaca fascicularis* | Body fluid contact | (80) |
| Borna virus |  | *Macaca fuscata* | *Macaca fuscata* |  | (224) |
| *Cercopithecine herpesvirus 1* | *Macaca fascicularis*  *Macaca mulatta*  *Macaca thibetana* | *Macaca fascicularis* | *Rhinopithecus roxellanae* |  | (115)(116)(119)  (120)(121) |
| Epstein-Barr |  |  | *Pongo pygmaeus* |  | (95) |
| Mumps |  |  |  |  |  |
| Foamy virus |  |  |  |  |  |
| Golden snub nosed monkey cytomegalovirus |  |  | *Rhinopithecus roxellanae* |  | (119) |
| Hepatitis B virus | *Macaca fascicularis* |  |  |  | (122) |
| Rhesus cytomegalovirus | *Macaca mulatta* |  |  |  | (116) |
| Simian Foamy virus | *Macaca fascicularis*  *Macaca mulatta*  *Macaca thibetana* |  | *Macaca mulatta*  *Rhinopithecus roxellanae* |  | (115)(116)(117)  (118)(119)(122) |
| Simian Pox virus | *Macaca thibetana* |  |  |  | (115) |
| Simian Retrovirus |  |  |  |  |  |
| Simian Type D Retrovirus | *Macaca mulatta* |  |  |  | (116) |
| Simian virus 40 |  |  |  |  |  |
| Simian T-cell Lymphotropic virus | *Macaca mulatta*  *Macaca thibetana* |  |  |  | (115)(116) |
| Adenovirus | *Macaca fascicularis* |  | *Pongo sp*  *Rhinopithecus roxellanae* | Respiratory | (95)(119)(225)  (226) |
| Calicivirus |  |  | *Macaca maura* |  | (195) |
| Influenza virus | *Macaca fascicularis*  *Macaca nemestrina* |  | *Macaca fascicularis* |  | (227) |
| Influenza A | *Macaca tonkeana* |  |  |  | (196) |
| *Measles morbillivirus* | *Macaca tonkeana*  *Macaca mulatta* |  | *Macaca mulatta* |  | (196)(197) |
| ParaInfluenza 1 | *Macaca tonkeana* |  |  |  | (196) |
| ParaInfluenza 2 |  |  |  |  |  |
| ParaInfluenza 3 |  |  |  |  |  |
| Respiratoy syncytial |  |  | *Pongo pygmaeus* |  | (95) |
| Astrovirus | *Macaca fascicularis* |  | *Macaca mulatta* |  | (225)(228) |
| Coxsackie B-4 |  |  | *Pongo pygmaeus* | Fecal/Oral | (95) |
| Enterovirus | *Macaca fascicularis*  *Macaca mulatta*  *Semnopithecus entellus*  *Trachypithecus pileatus* |  | *Macaca mulatta*  *Semnopithecus entellus*  *Trachypithecus pileatus* |  | (198)(225)(229)  ) |
| Hepatitis A | *Macaca thibetana* |  | *Macaca fascicularis*  *Rhinopithecus roxellanae* |  | (115)(119)(199) |
| Parechovirus | *Macaca mulatta*  *Semnopithecus entellus*  *Trachypithecus pileatus* |  | *Macaca mulatta*  *Semnopithecus entellus*  *Trachypithecus pileatus* |  | (198) |
| Parvovirus |  |  | *Macaca maura* |  | (195) |
| Picornavirus |  |  | *Macaca maura* |  | (195) |
| Primate Bocaparvovirus Species 3 |  |  | *Macaca mulatta* |  | (172) |
| Rotavirus A | *Macaca mulatta* | *Macaca mulatta* |  |  | (230) |
| Rotavirus SA11 |  |  | *Pongo pygmaeus* |  | (95) |
| Bunyavirus |  |  | *Macaca sinica* |  | (128) |
| Chikungunya virus |  | *Macaca fascicularis* | *Macaca fascicularis*  *Macaca mulatta*  *Macaca leonina*  *Macaca nemestrina*  *Macaca arctoides* | Vector Borne | (124)(126)(231) |
| Dengue virus |  | *Pongo pygmaeus* | *Macaca fascicularis*  *Macaca leonina*  *Macaca arctoides*  *Macaca sinica*  *Pongo pygmaeus* |  | (95)(123)(124)  (125)(128) |
| Flavivirus |  |  | *Macaca maura* |  | (195) |
| *Japanese encephalitis* | *Macaca fascicularis* | *Macaca fascicularis*  *Pongo pygmaeus* | *Pongo pygmaeus*  *Macaca fascicularis*  *Macaca mulatta*  *Macaca nemestrina* |  | (95)(123)(126)  (127)(129) |
| Kyasanur Forest Disease |  |  | *Macaca radiata*  *Semnopithecus entellus*  *Trachypithecus pileatus* |  | (200)(201) |
| Sindbis virus |  | *Pongo pygmaeus* | *Pongo pygmaeus* |  | (95)(123) |
| Tembusu virus |  |  |  |  |  |
| West Nile virus |  |  | *Macaca fascicularis* |  | (202) |
| Zika virus | *Macaca fascicularis* | *Pongo pygmaeus* | *Macaca fascicularis*  *Macaca leonina*  *Macaca sinica*  *Pongo pygmaeus* |  | (95)(123)(124)  (232) |

**Supplementary Table S5 :** List of zoonotic viruses found in each habitat type defined by color (blue = urban, orange = rural, green = forest). Each colored box represents the presence of a pathogen in an interface.

**Table S6** : Distribution in primate hosts of zoonotic Bacteria in interface habitats and their transmission routes

|  | Urban habitat | Rural habitat | Forest habitat | Main transmission route | References |
| --- | --- | --- | --- | --- | --- |
| *Mycobacterium tuberculosis* | *Macaca mulatta*  *Macaca fascicularis* | *Macaca mulatta* |  | Respiratory | (143) |
| *Streptococcus sp* | *Macaca fascicularis* |  |  |  | (144) |
| *Streptococcus equi zooepidemicus* | *Macaca fascicularis* |  |  |  | (145)(146) |
| *Bartonella quintana* | *Macaca fascicularis*  *Macaca fuscata* |  |  | Vector Borne | (140)(141) |
| *Candidatus Mycoplasma haemomacaque* | *Macaca fascicularis* |  |  |  | (142) |
| *Burkholderia pseudomallei* | *Macaca fascicularis* |  | *Macaca fascicularis* | Fecal/Oral | (82)(203) |
| *Campylobacter coli* | *Macaca sinica* |  |  |  | (204) |
| *Campylobacter helveticus* | *Macaca mulatta* |  |  |  | (205) |
| *Campylobacter jejuni* | *Macaca sinica*  *Macaca mulatta* |  | Macaca mulatta |  | (79)(204) |
| *Candidatus Campylobacter infans* | *Macaca mulatta* |  |  |  | (205) |
| *Citrobacter sp* | *Macaca fascicularis* |  |  |  | (135) |
| *Enterococci faecalis* | *Macaca mulatta* |  | *Macaca mulatta* |  | (233) |
| *Enterococci faecium* | *Macaca mulatta* |  | *Macaca mulatta* |  | (233) |
| *Enterococcus sp* | *Macaca fascicularis*  *Trachypithecus cristatus* |  | *Trachypithecus cristatus* |  | (137) |
| *Enterobacter sp* | *Macaca fascicularis* |  | *Macaca fascicularis* |  | (135) |
| *Escherichia coli* | *Macaca fascicularis*  *Macaca mulatta* | *Macaca mulatta*  *Macaca fuscata* | *Macaca mulatta* |  | (37)(79)(134)  (135) |
| *Hafnia paralvei* | *Macaca mulatta* |  | *Macaca mulatta* |  | (79) |
| *Klebsiella pneumoniae* |  |  |  |  |  |
| *Leptospira sp* | *Macaca mulatta*  *Macaca fascicularis* |  |  |  | (79)(203) |
| *Leptospira interrogans* |  |  | *Pongo pygameus* |  | (95) |
| *Mycobacterium avium paratuberculosis* | *Macaca mulatta* | *Macaca mulatta* |  |  | (234) |
| *Proteus sp* | *Macaca fascicularis* |  |  |  | (135) |
| *Pasteurella multocida* | *Macaca mulatta* |  | *Macaca mulatta* |  | (79) |
| *Salmonella sp* | *Macaca fascicularis*  *Macaca mulatta* | *Macaca mulatta* | *Macaca mulatta* |  | (79)(135)(206) |
| *Salmonella virchow* | *Macaca sinica* |  |  |  | (204) |
| *Shigella sp* | *Macaca mulatta* | *Macaca mulatta* | *Macaca mulatta* |  | (37)(79) |
| *Staphylococcus sp* | *Macaca mulatta* | *Macaca mulatta* | *Macaca mulatta* |  | (206) |
| *Staphylococcus aureus* | *Macaca fascicularis*  *Macaca mulatta*  *Trachypithecus cristatus* | *Macaca mulatta* | *Macaca fascicularis*  *Macaca mulatta*  *Trachypithecus cristatus* |  | (79)(136)(137)  (138)(139) |
| *Streptococcus pneumoniae* | *Macaca mulatta* |  | *Macaca mulatta* |  | (79) |
| *Yersinia sp* | *Macaca mulatta* |  | *Macaca mulatta* |  | (79) |

**Supplementary Table S6 :** List of zoonotic bacteria found in each habitat type defined by color (blue = urban, orange = rural, green = forest). Each colored box represents the presence of a pathogen in an interface.
